# Bdf1 Bromodomains Are Essential for Meiosis and the Expression of Meiotic-Specific Genes

**DOI:** 10.1371/journal.pgen.1006541

**Published:** 2017-01-09

**Authors:** Encar García-Oliver, Claire Ramus, Jonathan Perot, Marie Arlotto, Morgane Champleboux, Flore Mietton, Christophe Battail, Anne Boland, Jean-François Deleuze, Myriam Ferro, Yohann Couté, Jérôme Govin

**Affiliations:** 1 U1038, Université Grenoble Alpes, Institut de Biosciences et Biotechnologies de Grenoble (BIG), Grenoble, France; 2 Laboratoire Biologie à Grande Echelle (BGE), Commissariat à l'énergie atomique et aux énergies alternatives (CEA), Institut de Biosciences et Biotechnologies de Grenoble (BIG), Grenoble, France; 3 U1038, Institut national de la santé et de la recherche médicale (Inserm), Grenoble, France; 4 Centre National de la Recherche Scientifique (CNRS), Institut de Biosciences et Biotechnologies de Grenoble (BIG), Grenoble, France; 5 Centre National de Génotypage, Institut de Génomique, Commissariat à l'énergie atomique et aux énergies alternatives, Evry, France; SUNY Stony Brook, UNITED STATES

## Abstract

Bromodomain and Extra-terminal motif (BET) proteins play a central role in transcription regulation and chromatin signalling pathways. They are present in unicellular eukaryotes and in this study, the role of the BET protein Bdf1 has been explored in *Saccharomyces cerevisiae*. Mutation of Bdf1 bromodomains revealed defects on both the formation of spores and the meiotic progression, blocking cells at the exit from prophase, before the first meiotic division. This phenotype is associated with a massive deregulation of the transcription of meiotic genes and Bdf1 bromodomains are required for appropriate expression of the key meiotic transcription factor *NDT80* and almost all the Ndt80-inducible genes, including APC complex components. Bdf1 notably accumulates on the promoter of Ndt80 and its recruitment is dependent on Bdf1 bromodomains. In addition, the ectopic expression of *NDT80* during meiosis partially bypasses this dependency. Finally, purification of Bdf1 partners identified two independent complexes with Bdf2 or the SWR complex, neither of which was required to complete sporulation. Taken together, our results unveil a new role for Bdf1 –working independently from its predominant protein partners Bdf2 and the SWR1 complex–as a regulator of meiosis-specific genes.

## Author Summary

Chromatin modifying proteins play a central role in transcription regulation and chromatin signalling. In this study we investigated the functional role of the bromodomains of the chromatin protein Bdf1 during yeast gametogenesis. Our results show that the bromodomains of Bdf1 are essential for meiotic progression and the formation of mature spores. Bdf1 bromodomains are required for the expression of key meiotic genes and the master regulator *NDT80*. Forced expression of *NDT80* can partially rescue the formation of spores when Bdf1 bromodomains are mutated. The results presented here indicate that Bdf1 forms two exclusive complexes, with Bdf2 or with the SWR complex. However, none of these complexes are required for sporulation progression. To conclude, our findings suggest that Bdf1 is a new regulator of the meiotic transcription program and of the expression of the master regulator *NDT80*.

## Introduction

Protein members of the BET family share a conserved modular architecture, with two bromodomains in their N-terminal part, an extra-terminal recruitment (ET) domain and other conserved motifs. Bromodomain modules bind acetylated lysines in histones and other proteins, and BET bromodomains specifically recognise the acetylated lysines of core histones, in particular H3 and H4 [1].

BET proteins are present in unicellular eukaryotes such as the model organism *Saccharomyces cerevisiae*, where two homologous genes, Bdf1 and Bdf2, are expressed. Bdf1 has a typical BET protein structure, with two bromodomains, one ET domain and other conserved motifs. Multiple functional roles have been proposed for this protein, which was first described as a regulator of snRNA [2] and then identified as part of the yeast’s general transcription factor, TFIID [3]. In yeast, unlike its human homologue TAF(II)250, the TAF(II)145 protein lacks a module with bromodomains. Bdf1 could thus represent the missing piece of this yeast TAF complex [3,4]. Moreover, Bdf1 plays a role in regulating gene expression in response to various stresses [5–8].

Bdf1 has been shown to be part of the SWR complex, which is responsible for the incorporation of the histone variant H2A.Z into chromatin [9,10]. Bdf1 is not required for the enzymatic activity of Swr1 but facilitates H2A.Z incorporation *in vivo* [5,11,12]. Two studies demonstrated that Bdf1 bromodomains are functional and bind multi-acetylated histones, where they mediate an anti-silencing function and prevent the spreading of Sir proteins at heterochromatin boundaries [13,14]. Unexpectedly, a high throughput study also implicated Bdf1 in pre-mRNA splicing [15]. Thus, this protein could potentially link chromatin remodelling, transcription initiation and pre-mRNA splicing.

Most of the data describing the functions of Bdf1 were obtained using vegetative *S*. *cerevisiae* cells. However, Bdf1 was described to play an essential role for sporulation when its gene was first identified in yeast [16]. In this process, nutrient starvation triggers a specific differentiation program in diploid *S*. *cerevisiae*, starting with meiosis and ending with the formation of four quiescent spores [17,18]. In their seminal work, Chua et al. [16] identified Bdf1 as a chromosomal protein with two bromodomains which was required for meiosis progression. We also found that spore chromatin is highly compacted, hyperacetylated, and enriched in Bdf1 [19]. Altogether, this information suggests that Bdf1 plays a functional role during the meiotic and post-meiotic stages of sporulation.

In mammals, Brd2, Brd3 and Brdt play specific roles in cellular differentiation in a range of tissues [20–22]. Thus, Brd2 is involved in neuronal development and differentiation [23,24], while Brd3 is essential to erythropoiesis [25]. Brdt is only expressed in the testis and regulates the expression program of meiotic and post-meiotic genes, driving the transcription program throughout sperm differentiation [26]. In addition, it is a key player in the final chromatin reorganisation step found in the final spermatic structure [27]. Brdt preferentially binds to hyperacetylated histones and promotes a drastic chromatin reorganisation during sperm differentiation [28,29]. Brd4 is also involved in regulating transcription during post-meiotic sperm differentiation and final chromatin reorganisation [30].

Altogether, BET proteins are important for gametogenesis. The role of Bdf1 during sporulation was the focus of the study presented here. Our results indicate that, although Bdf1 bromodomains are not required for growth on non-fermentable carbon sources, they are essential for sporulation to complete. Transcriptomic analysis during sporulation revealed that Bdf1 bromodomains play an essential coordinating role in the transcriptional program of meiosis leading to the formation of spores. In particular, Bdf1 was found to accumulate on the promoter of the master regulator *NDT80* before meiosis. This recruitment requires functional Bdf1 bromodomains and is essential for a normal expression of *NDT80*. Thus the ectopic expression of Ndt80 can partially overcome the defects observed when Bdf1 bromodomains are mutated. Finally, Bdf1 forms two exclusive complexes, neither of which was required for meiotic gene regulation or to complete sporulation.

## Results

### Specificity of Bdf1 bromodomains towards acetylated histones

#### Bdf1 bromodomains recognise multi-acetylated H4 *in vitro*

**Fig 1:**
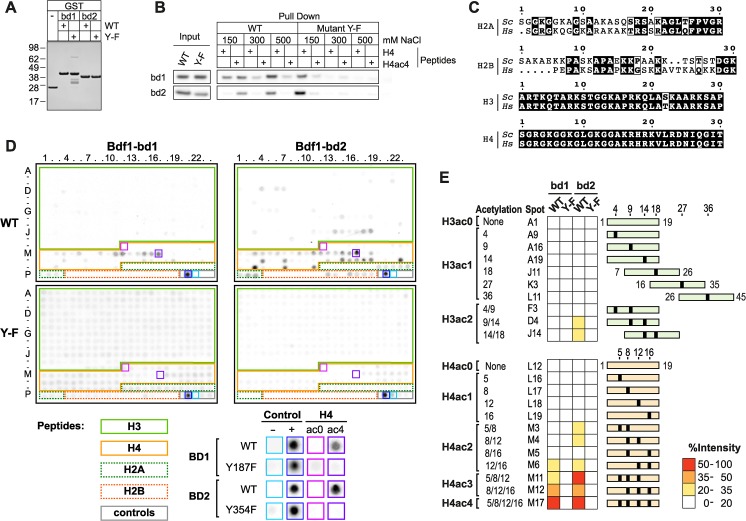
Bdf1 bromodomains bind multi-acetylated H4 tail. (A) Purified Bdf1 bromodomains (Coomassie stained gel). Wild-type (WT). Mutations are Y187F and Y354F for Bdf1 bromodomain 1 and 2, respectively. (B) Pull-down assay using histone H4 (H4) and tetra-acetylated H4 peptides (H4K5ac K8ac K12ac K16ac, H4ac4). (C) Sequence alignment of the N-terminal sequences of H2A, H2B, H3 and H4 from human (Hs) and *S*. *cerevisiae* (Sc). (D) Binding profiles on a histone peptide array for WT and Y-F mutant of Bdf1 bromodomains. Control, H2A, H2B, H3 and H4 peptides are highlighted in grey, dashed green, dashed orange, plain green, plain orange, respectively. Signal for background, positive control, H4ac0 and H4ac4 peptides are highlighted on the array and shown at a higher magnification below the array. Signal intensity data can be found in S1 Table. (E) Bdf1 bromdomain binding intensities for a selection of H3 and H4 peptides. Acetylated sites are represented by black boxes on green (H3) or orange (H4) rectangles.

The specificity of Bdf1 bromodomains has been analysed with recombinant bromodomains (BD, Fig 1A). The functionality of these recombinant BD1 and BD2 domains was confirmed by control pull-down assays performed with H4 and tetra-acetylated H4 peptides (Fig 1B). Tyrosine residues 187 and 354 are essential for the functionality of Bdf1 bromodomains (S1A Fig, [14]). As expected, their mutation into a phenylalanine (Y-F) abolishes detectable binding of the BDs to either the acetylated or non-acetylated peptides (Fig 1B). Interestingly, additional pull-down experiments showed that Bdf1 bromodomain 2 has a lower affinity for H4 acetylated peptide than human Brd4 BD1 (S1B Fig). This could be explained by the fact that tyrosine 338 in *S*. *cerevisiae* is not conserved in human BET proteins, where this position is occupied by a tryptophan residue (S1A Fig). In support of this hypothesis, mutation of tyrosine 338 to tryptophan in Bdf1 bromodomain 2 dramatically increased its binding to acetylated H4 (S1B Fig); whether this difference in affinity has any functional role *in vivo* remains to be determined.

The diversity of potential ligands of Bdf1 bromodomains was then explored using commercial peptide arrays of ~400 peptides mimicking modified residues in human core histones. While the H2A and H2B sequences diverge considerably between human and *S*. *cerevisiae*, the H3 and H4 sequences are almost identical, thus this array is appropriate for the study of these histones (Fig 1C). The salt concentrations optimized for the pull-down assays were used to give the best signal-to-noise ratio on the peptide arrays (Fig 1B, 300 mM NaCl for Bdf1-BD1 and 500 mM for Bdf1 BD2). Signals obtained with negative (mock) and positive controls (myc peptide) as well as the unmodified and tetra-acetylated H4 peptides were consistent with the results of the pull-down experiments (Fig 1B and 1D). Moreover, the Y-F mutation in BD1 and BD2 abolished binding to modified peptides, thus confirming the specificity of the interactions observed with the WT constructs on the peptide arrays (Fig 1D). Array signals were then quantified and normalised relative to the intensity of the positive control (myc peptide, data are presented in S1 Table). For both Bdf1 bromodomains, the strongest binding was detected with a H4 peptide tetra-acetylated on lysines 5, 8, 12 and 16 (H4ac4, Fig 1E), in line with previously published data from experiments using a recombinant protein containing both Bdf1 bromodomains [13, 14]. Our result suggests some redundancy in the ligand-binding affinity of both Bdf1 bromodomains. This redundancy contrasts with the affinities of the BET proteins Brd2 and Brdt bromodomains, which are specific for different acetylated sites [1,31].

#### Both Bdf1 bromodomains are required for H4ac4 recognition *in vivo*

**Fig 2:**
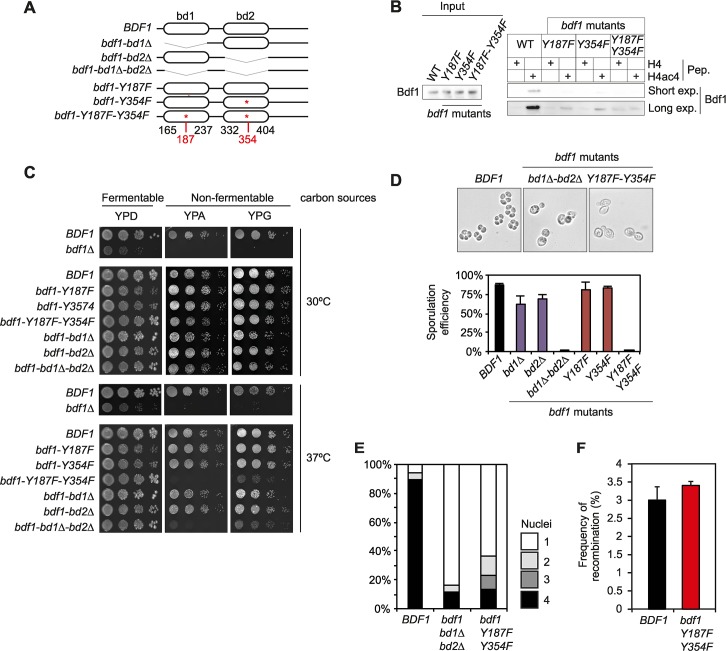
Bdf1 bromodomains are essential for sporulation. (A) Schematic representation of the different *bdf1* mutants. Bromodomains were deleted or mutated individually or in combination. Limits of bromodomains were based on [8]. Point mutations are identical to those used in Fig 1. (B) Pull-down analysis on H4 peptides (H4K5ac K8ac 12ac 16ac, H4ac4) using whole cell extracts from different *bdf1* mutant strains. Bdf1 was detected using an antiserum specifically developed for this study (S4A Fig). Two exposure times, short or long, are presented and labelled "Short Exp" and "Long Exp", respectively. (C) Growth assay with *bdf1* mutants on fermentable (glucose, YPD) and non-fermentable carbon sources (acetate or glycerol, YPA or YPG respectively). Assays were performed at two temperatures, 30°C and 37°C. (D) Sporulation efficiency of the Bdf1 bromodomain mutants. Mutation of both bromodomains of Bdf1 hampers the formation of spores (top). Quantification of sporulation efficiency (bottom). (E) Analysis of meiotic divisions by DAPI staining in *bdf1* mutant strains. (F) Analysis of meiotic recombination in the *bdf1-Y187F-Y354F* mutant using the heteroalleles *his4N / his4G*.

Random transposon insertion identified Bdf1 as essential for sporulation [16]. In addition, the insertion of transposons inside the *BDF1* ORF induced sporulation defects only when the second bromodomain of Bdf1 was targeted [16]. For this study, we created a collection of mutant strains in which each bromodomain was deleted or mutated (Y-F mutation as described above, Fig 2A). The functionality of each construct was then tested by pull-down using whole cell extracts. As expected, WT Bdf1 specifically bound tetra-acetylated H4 peptides (Fig 2B). Point mutation in a single bromodomain did not totally abolish Bdf1 binding to acetylated H4 peptides, even though the signal was clearly decreased compared to the WT protein (Fig 2B). However, mutation of both bromodomains completely abolished the specific binding of Bdf1 to the H4ac4 peptide. Therefore, it appears that two functional bromodomains are necessary to maintain strong recognition of acetylated H4 by the Bdf1 protein *in vivo*.

### Functional role of Bdf1 bromodomains *in vivo*

#### Bdf1 bromodomains are not required for growth on non-fermentable carbon sources

Several studies have already examined the requirement for Bdf1 to maintain growth on non-fermentable carbon sources [2,8,16,32]. We confirmed that *BDF1* is essential for growth at 30°C on non-fermentable carbon sources such as acetate and glycerol (Fig 2C). However, this phenotype does not depend on the functionality of Bdf1 bromodomains: their deletion / mutation does not affect yeast growth, even when both are deleted. However, a strain deleted or mutated for both bromodomains loses its capacity to grow on non-fermentable carbon sources at 37°C (Fig 2C). The Bdf1 protein has been suggested to be involved in mitochondrial function [8] and the fact that Bdf1 bromodomains are not required for the use of non-fermentable carbon sources could be related to a partial redundancy with *BDF2* (see below).

#### Bdf1 bromodomains are required for sporulation

Sporulation is induced when diploid yeasts are deprived of a fermentable carbon source and of nitrogen. Deletion of one bromodomain, either BD1 or BD2, reduced the efficiency of spore formation by 30% compared to a WT strain (Fig 2D); this phenotype was less pronounced when bromodomains were disrupted by point mutations. In contrast, deletion or mutation of both Bdf1 bromodomains abolished spore formation (Fig 2D). DAPI staining revealed that progression of meiosis was impaired when both Bdf1 bromodomains were non-functional (Fig 2E). This result indicates that Bdf1 bromodomains are specifically required during the interval between the pachytene phase and the first meiotic division.

Then, the effect of the mutation of Bdf1 bromodomains on meiotic recombination was tested using the heteroalleles *his4-N/his4-G*. In this assay, germination of wild-type spores on a medium without histidine (SC-HIS) was used to determine the frequency of formation of a wild type *HIS4* allele by recombination between the *his4-N/his4-G* heteroalleles. Even if most of the *bdf1-Y187F-Y354F* cells are stalled in meiosis after induction of sporulation, they can escape this arrest and return to vegetative growth if they are placed in rich medium. This escape pathway is called "return-to-growth" [33] and enabled the quantification of *HIS4* meiotic recombination in the *bdf1-Y187F-Y354F* mutant, even when no spores were formed. Altogether, comparable rates of recombination appear were measured in the wild-type and Bdf1 bromodomains mutants (Fig 2F). This result corroborates the phenotype described for a full length deletion of *BDF1* [16].

### Bdf1 bromodomains regulate the meiotic transcription program

Deregulation of some master regulators of sporulation could be an obvious explanation for failure to induce this differentiation program. The expression of early genes *IME1* and *IME2*, which is required for progression through meiotic S and G2 phases, was not significantly affected by mutation of the Bdf1 bromodomains (S2A Fig). Middle and late sporulation genes are essential for meiotic divisions and post-meiotic spore differentiation. The expression of a selection of these genes was tested by RT-qPCR and found to be defective when Bdf1 bromodomains are mutated (S2B and S2C Fig). This result is consistent with the sporulation phenotype of the strain, which is blocked at the transition between the pachytene phase and meiotic divisions.

**Fig 3:**
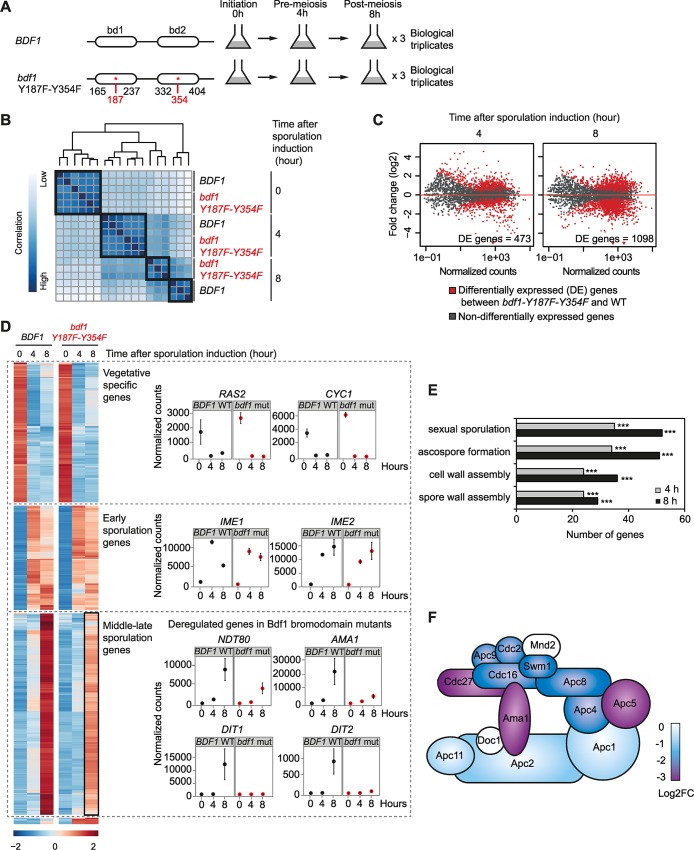
Bdf1 bromodomains regulate the meiotic transcription program. (A) Experimental design for RNA-seq experiments. (B) Unsupervised hierarchical clustering of the distance between samples. All replicates are presented. (C) MA-plots representing the mean normalised counts for each gene in the WT sample at different times after the induction of sporulation (x-axis). Fold change in expression levels when comparing the WT and *bdf1-Y187F-Y354F* strains are represented on the y-axis (log2). Differentially expressed (DE) genes (fold change below -2 or above 2, adjusted p-value < 0.05) are represented in red and their number is indicated at each time-point. Details of these genes can be found in S5 Table. (D) Clustering of the most variable genes during sporulation (left). Clusters were categorized based on the sporulation transcription program (left) and are presented with the expression levels of representative vegetative, early and middle genes (right). (E) Functional enrichment analysis of GO-defined biological processes among differentially expressed genes. The number of genes differentially expressed for each GO term is represented on the x-axis at 4 h (grey) and 8 h (black). The symbol *** indicates a p-value < 0.001. (F) Schematic representation of the APC complex subunits colour-coded to represent their fold change between WT and Bdf1 bromodomain mutants.

RNA-seq experiments provided a general view of the transcriptional defects caused by mutation of both Bdf1 bromodomains. The expression of *BDF1* was monitored in triplicate experiments performed on the WT and in *bdf1-Y187F-Y354F* strains at three time-points (before sporulation induction (0 h), 4 and 8 hours after induction, Fig 3A). The bioinformatics pipeline used in the analysis of the results of these experiments is presented in S3A Fig. Increased *BDF1* transcript and protein levels were detected when its bromodomains were mutated, possibly as part of a compensatory response by the cell (S3B Fig). Furthermore, our results confirmed that Bdf1 regulates *BDF2* mRNA levels, particularly during sporulation when the mutation of Bdf1 bromodomains may induce a compensatory overexpression of *BDF2* (S3B Fig, [34]).

The distance between each replicate was analysed and, as expected, biological replicates appeared clustered by strain and sporulation time-point (Fig 3B). Thus, before sporulation was induced, the transcription program was similar in the WT and *BDF1* bromodomain mutant strains. Defects started to accumulate from 4 h and culminated at 8 h when the *BDF1* bromodomain mutant strain stopped progressing through the sporulation program (Fig 3B and 3C). The same observation was made based on an independent assessment using principal component analysis (S3C Fig).

#### Bdf1 bromodomains are not required for pre-mRNA splicing

Bdf1 was previously identified as important for pre-mRNA splicing [15,34]. Normalized counts for each intron were compared in the WT and *bdf1-Y187F-Y354F* strain in vegetative cells (S3D Fig). No significant difference was found on five intron containing genes, which were previously used as reporters for splicing efficiency [15]. In addition, no difference was found when considering all introns, in contrast to the defects observed in the *bdf1*Δ strain (S3D Fig, "all introns" and ref [15]).

#### Analysis of differentially expressed genes

The gene expression program was first examined during sporulation in the WT strain using the DESeq2 R-package, with an adjusted p-value less than 0.05 and a fold change below -2 or above 2. Results of this analysis were in agreement with previously published data [35–37]. Four hours after induction, 267 genes were significantly upregulated and 206 were significantly downregulated; after 8 hours, 343 genes were upregulated and 755 downregulated (S5 Table). Representative downregulated genes at 4 h and 8 h are presented Fig 3D.

The list of differentially expressed genes between the WT and the *bdf1-Y187F-Y354F* mutant strain was established for each time-point using the DESeq2 R-package (Fig 3C, S5 Table, p-value < 0.05 and |Fold change| > 2). Of the genes analysed, none showed a statistically significant difference in expression before sporulation induction between the WT and *bdf1-Y187F-Y354F* strains. The same analysis was performed on the data obtained 4 and 8 hours after inducing sporulation; in these conditions large numbers of deregulated genes were identified, 473 and 1,098, respectively (Fig 3C and 3D, S5 Table). Notably, *NDT80*, *AMA1* and late genes such as *DIT1* and *DIT2* failed to induce when Bdf1 bromodomains were mutated (Fig 3D).

GO term analysis of genes differentially expressed during sporulation identified defects in groups of genes involved in sexual sporulation, ascospore formation, cell and spore wall formation (Fig 3E). In addition, analysis of protein complex enrichment identified a defect in the expression of 11 of the 13 subunits of the APC complex (Fig 3F) and of *AMA1*, an activator of the APC complex. *AMA1* has an intron and its splicing was not affected during sporulation (S3E Fig).

### Mutations of Bdf1 bromodomains impair expression of *NDT80* and Ndt80-regulated genes

**Fig 4:**
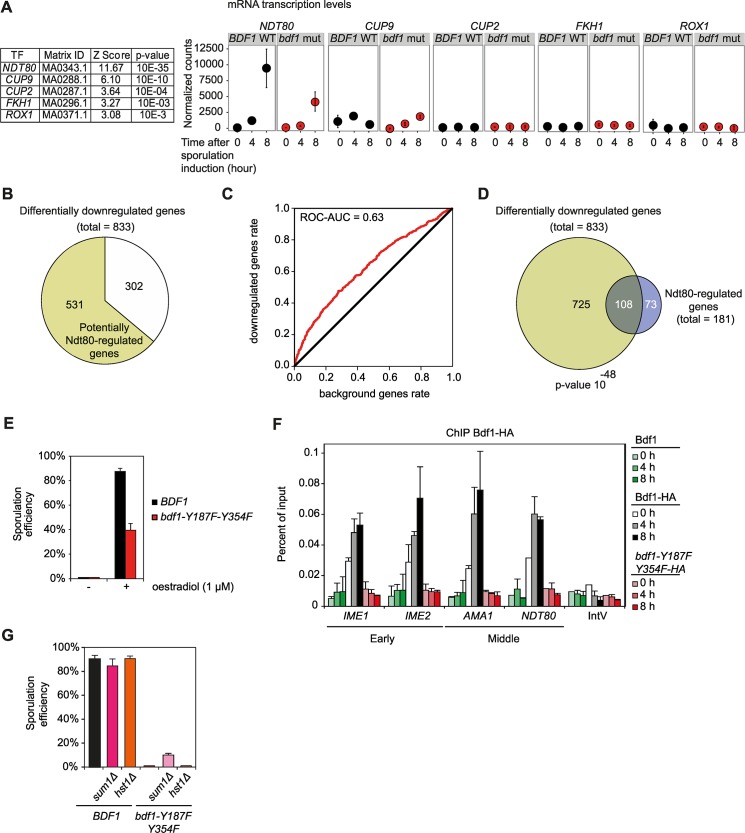
Bdf1 is required for the transcription of *NDT80* and middle sporulation genes. (A) List of over-represented transcription factors with binding sites in the promoters of the genes which are differentially downregulated at 4h and 8h when Bdf1 bromodomains are mutated (fold change < -2, adjusted p value < 0.05). A transcription factor was considered significantly over-represented when the p-value describing its enrichment was below 0.001. On the right, the normalized counts present the expression levels for *NDT80*, *CUP9*, *CUP2*, *FKH1* and *ROX1*. (B) Pie chart representing the proportion of genes which are downregulated in the *bdf1-Y187F-Y354F* strain at 4 and 8h of sporulation induction (833 genes in total) and with putative Ndt80 binding sites in their promoter (531 genes, their list is available in S5 Table). (C) ROC-AUC plot representing the enrichment of the Ndt80 binding sequence in the promoter of the genes downregulated in the *bdf1-Y187F-Y354F* mutant strain. (D) Venn diagram illustrating the overlap between genes differentially downregulated in *bdf1* bromodomain mutant at 4 and 8h of sporulation induction (833 genes) and genes previously identified as Ndt80-regulated in Chu *et al*. [35]. (E) Sporulation efficiency upon ectopic expression of *NDT80* in WT and *bdf1-Y187F-Y354F* strains during sporulation. Here, the endogenous *NDT80* promoter was replaced by an oestrogen-dependent promoter and its expression activated by the addition of oestradiol 6 hours after sporulation induction [44,45]. (F) Bdf1 chromatin immunoprecipitation. Bdf1 occupancy was monitored in the meiotic-specific promoters of the early genes *IME1* and *IME2* and middle genes *AMA1* and *NDT80* during sporulation. Mutation of Bdf1 bromodomains impairs the recruitment of Bdf1 to chromatin. IntV is an intergenic and transcriptionally inactive region of chromosome V between coordinates 9,762 and 9,812. (G) Sporulation efficiency when Bdf1 bromodomain mutations are combined with Sum1 and Hst1 deletions, two known repressors of *NDT80* activation [41].

A total of 833 genes were differentially downregulated when combined between the 4h and 8h time points. The examination of their promoters revealed that binding sequences of the transcription factors *NDT80*, *CUP9*, *CUP2*, *FKH1* and *ROX1* were statistically enriched (p-value <0.001, Fig 4A). Of these, only Ndt80 has been described as essential for sporulation and is known to be a key meiotic regulator [38–41]. Indeed, Ndt80 is induced during meiosis where it is expressed at much higher levels than Cup9, Cup2, Fkh1 and Rox1 (Fig 4A). Defects in Ndt80 expression also explain the non-induction of middle sporulation genes, such as *SMK1* and *SSP1*, as expression of these genes is controlled by Ndt80; both were downregulated in the bromodomain mutant strain (S2B Fig, [35,42]).

Ndt80 is the key transcription factor that activates the promoters of middle sporulation genes and is expressed just before the middle genes are induced [41]. The examination of the promoters of the 833 differentially downregulated genes identified 531 genes with putative *NDT80* binding sites (Fig 4B, S5 Table). In addition, the probability that an Ndt80 binding sequence is present within the promoter of Bdf1-regulated genes was calculated based on the ROC-AUC value produced by the MORPHEUS webtool [43] (Fig 4C). The ROC-AUC value of 0.63, which is above 0.5, indicates an enrichment of the Ndt80 binding sequence in the genes regulated by Bdf1 bromodomains when compared to a list of unrelated genes. Finally, a list of genes potentially regulated by Ndt80 was obtained from Chu et al [35]. These genes were statistically over-represented in the list of genes downregulated in the *bdf1-Y187F-Y354F* mutant (Fig 4D, p-value = 10^−48^ with a hypergeometric test). In conclusion, a significant proportion of the genes which fail to induce when Bdf1 bromodomains are mutated are regulated by Ndt80.

An oestradiol-inducible form of *NDT80* was used to test whether a reduction in Ndt80 levels could explain the phenotype observed when Bdf1 bromodomains are mutated [44,45]. This system was introduced into the *bdf1-Y187F-Y354F* mutant and oestradiol was added 6 hours after sporulation induction. Induction of *NDT80* expression at this stage partially alleviated the defects observed when Bdf1 bromodomains are mutated, with 40% of spores formed in this mutant strain (Fig 4E). This result indicates that mutation of Bdf1 bromodomains affects the expression of the master regulator *NDT80* alters the expression of middle sporulation genes and that these sporulation defects can be partly rescued by ectopic overexpression of *NDT80*.

### Bdf1 bromodomains bind to the *NDT80* promoter to promote its transcriptional activation

#### Bdf1 is recruited to the NDT80 promoter before its activation

Bdf1 and other BET proteins promote transcription activation [2,46–48]. We performed chromatin immunoprecipitation (ChIP) experiments to monitor Bdf1 enrichment on the promoters of key sporulation genes. For these experiments, the Bdf1 protein was HA-tagged; this tag does not affect the progression of sporulation (S5A Fig). ChIP experiments detected Bdf1 on the promoters of *IME1*, *IME2*, *AMA1* and *NDT80* before sporulation (0 h, Fig 4F). Recruitment of Bdf1 to *IME1* and *IME2* promoters increased when these genes were activated 4 h after the induction of sporulation (*IME1* and *IME2*, 4 h, Fig 4F). The presence of Bdf1 on the *AMA1* and *NDT80* promoters was close to maximal 4 h after induction, before maximal transcription of these genes occurs (*AMA1* and *NDT80*, 4 h, Figs 3D and 4A and S2B). These observations suggest that Bdf1 is loaded on *AMA1* and *NDT80* promoters before the start of transcription and promote their subsequent activation. This recruitment is dependent on the Bdf1 bromodomains and is lost in the *bdf1-Y187F-Y354F* mutant strain (Fig 4F). Finally, Bdf1 is not detected in an inactive intergenic region (Fig 4F).

#### Known *NDT80* regulators and Bdf1 bromodomains

In vegetative cells and before exit of the pachytene, *NDT80* is repressed by the Sum1 factor, the histone deacetylase Hst1 and a third partner, Rfm1 (for review [41]). We tested whether deletion of *SUM1* or *HST1* could overcome the defects observed when Bdf1 bromodomains are mutated. 10% of spores formed when *SUM1* was deleted in the *bdf1-Y187F-Y354F* mutant (Fig 4G). The deletion of *HST1* did not alter the sporulation phenotype in cells where Bdf1 bromodomains were mutated (Fig 4G). Sum1 de-repression is thought to occur during meiotic G2, and this has been proposed to be a regulatory step that must occur before the *NDT80* positive autoregulatory loop can be engaged [41]. These results suggest that the reason *NDT80* mRNA levels are reduced in the Bdf1 mutant is that bromodomains are required as part of *NDT80*’s positive autoregulatory loop.

### Bdf1 predominantly interacts with Bdf2

**Fig 5:**
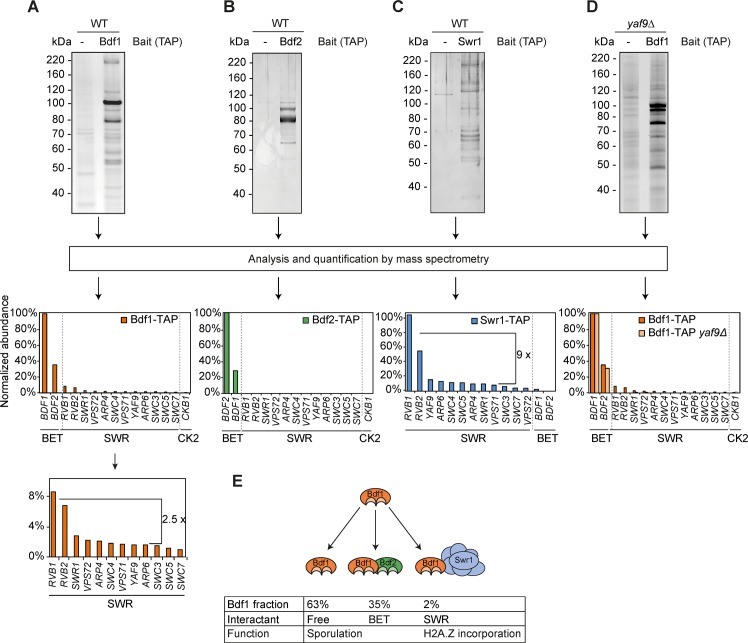
Bdf1 interactome during sporulation. Representative silver-stained gels of TAP purification eluates in vegetative cells of Bdf1 (A), Bdf2 (B), Swr1 (C), and Bdf1 in the absence of the subunit *YAF9* of the SWR complex (D). Interactants were identified and quantified by mass spectrometry. The relative abundances of the major partners of each bait are presented below each gel and in S6 Table. (E) Summary of the Bdf1 complexes. Bdf1 is mostly found as a free protein. It also forms two mutually exclusive complexes with Bdf2 or SWR complex.

We next wondered whether Bdf1 interactants could contribute to the regulation of the meiotic transcription program and mediate the transcriptional activation of *NDT80*. The interactome of Bdf1 during vegetative growth was determined using mass spectrometry-based proteomic analysis of affinity purified samples (Fig 5). The relative abundances of Bdf1 partners were determined using the mass spectrometry-based iBAQ metrics [49,50]. Interestingly, the most abundant Bdf1 partner was Bdf2. The 12 subunits of the SWR complex, involved in the incorporation of the yeast histone variant H2A.Z, were also identified [9]. Both of these interactions with Bdf1 were confirmed by western blot (S4B Fig). The relative stoichiometry of the SWR subunits found to interact with Bdf1-TAP was compatible with the recently published structure of the complex, where the subunits Rvb1/2 are 2.5 times more abundant than the other subunits (Fig 5A bottom, [51]). Finally, the kinase CK2 was also detected (Fig 5A and S6 Table). This protein had previously been associated with Bdf1 [9].

Additional purifications were then performed to refine our knowledge of how the Bdf1 interactome is organised. Bdf2-TAP purifications were performed and identified Bdf1 as the major partner (Fig 5B). None of the SWR subunits was associated with Bdf2.

The Swr1 protein was then TAP-tagged and its complex purified. This analysis identified all the subunits of the SWR complex (Fig 5C). Bdf1 was identified as a minor interacting partner of Swr1 compared to the well-established SWR complex subunits. In these experiments, a 9 fold difference in relative abundance was detected for Rvb1/2 and the other SWR subunits. This difference with the data obtained in the Bdf1 complex and the published structure of the SWR complex could be explained if the SWR complex is present *in vivo* in several forms. This hypothesis is strengthened by results from structural studies using cosslinking reagents to obtain a homogeneous population of the complex [51]. Interestingly, Bdf2 was never identified in SWR complex. This result suggests that Bdf1 could form two exclusive complexes, one with the SWR complex and another one with Bdf2.

Finally, Bdf1-TAP was purified from a strain lacking *YAF9*, the subunit of the SWR complex responsible for recruiting Bdf1 [52]. In the *yaf9*Δ strain, Bdf1 still associated with Bdf2, but its interaction with the SWR complex was completely abolished (Fig 5D). Taken together, these results suggest that Bdf1 can be part of two exclusive complexes: one with the SWR complex or a Bdf1 / Bdf2 complex whose function remains to be characterised (Fig 5E).

### Sporulation progression is independent of SWR complex

**Fig 6:**
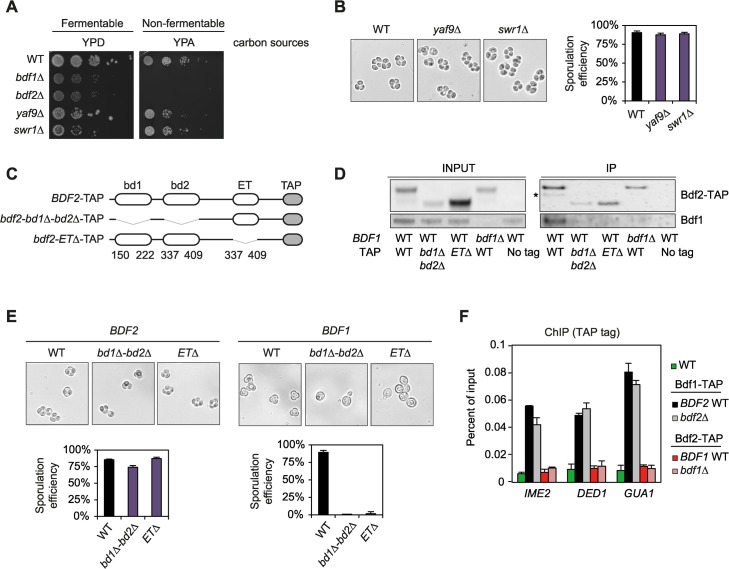
The function of Bdf1 during sporulation is independent of the complexes it forms with Bdf2 and the SWR complex. (A) Growth assay with WT, *bdf1Δ*, *bdf2Δ*, *yaf9Δ* and *swr1Δ* mutants on fermentable (glucose, YPD) and non-fermentable (acetate, YPA) carbon sources. (B) Sporulation efficiency of *yaf9*Δ and *swr1*Δ mutant strains. (C) Schematic representation of the different Bdf2 mutants in which bromodomains or the ET domain were deleted. All constructs are tagged with TAP. (D) Analysis of the interactions between Bdf2-TAP mutants and Bdf1. Bdf2-TAP was immunoprecipitated and the co-precipitation of Bdf1 was analysed by western blot using the Bdf1 antiserum (S4A Fig). The same membrane was then used to detect Bdf2-TAP (asterisk identifies co-precipitated Bdf1). (E) Sporulation efficiency of *BDF1* and *BDF2* mutants in which both bromodomains (*bd1Δbd2Δ)* or the ET domain (*ET*Δ) are deleted. Representative images and quantification data are shown. (F) Bdf1-TAP or Bdf2-TAP chromatin immunoprecipitation in WT, *bdf1*Δ and *bdf2*Δ.

The sporulation phenotypes induced by the mutations of Bdf1 bromodomains could be functionally explained by its interactions with the SWR complex or with Bdf2. To investigate this hypothesis, *YAF9* and *SWR1* were mutated in the SK1 genetic background and compared to *bdf1* mutant strains. Deletion of *YAF9* or *SWR1* had no effect on growth in any of these conditions (Fig 6A), nor did *YAF9* and *SWR1* deletions affect sporulation efficiency (Fig 6B). Therefore, the sporulation defects observed when Bdf1 bromodomains are mutated are not related to the function of the SWR complex.

In the absence of a role for the SWR complex in sporulation, we hypothesised that the Bdf1 / Bdf2 complex could play a role during sporulation which may be related to the defects observed when Bdf1 bromodomains are mutated. As previously published [2,8,16,32], strains deleted for *BDF1* and *BDF2* exhibit a slow growth phenotype on fermentable carbon sources, and fail to grow on non-fermentable carbon sources such as acetate or glycerol (Fig 6A). Different regions of Bdf2 were deleted to map the regions required for its interaction with Bdf1 (Fig 6C). This analysis indicated that Bdf2 bromodomains are essential for its interaction with Bdf1 (Fig 6D). Interestingly, deletion of the two Bdf2 bromodomains had no phenotypic effects either on non-fermentable carbon sources or during sporulation (Fig 6E, left). Finally, we studied the functional role of the Extra-Terminal domain (ET), which is a conserved protein–protein interacting domain that regulates transcriptional activity [53,54]. Deleting this domain in *BDF2* disrupted its interaction with Bdf1 (Fig 6D) but had no effect on sporulation (Fig 6E, left). Deletion of the same domain in Bdf1 did not affect growth on non-fermentable carbon sources at 30°C but revealed some defects at 37°C (S5B Fig). In contrast to Bdf2, the ET deletion in Bdf1 affects sporulation to a similar extent to the mutation of its bromodomains (Fig 6E, right).

These last results underline the fact that even though Bdf1 and Bdf2 are highly similar in their modular organisation, they play different roles during sporulation. Thus, only Bdf1 bromodomains, and not Bdf2 bromodomains, are essential for the formation of spores. Moreover, the sporulation defects observed in strains with mutated Bdf1 bromodomains are not mediated by any of its partners. Indeed, the disruption of the interaction of Bdf1 with Bdf2 or with the SWR complex generated no sporulation defects.

### Bdf1 is recruited to chromatin independently of Bdf2

Finally, we examined whether deletion of *BDF2* impacted Bdf1 recruitment to chromatin. ChIP experiments suggest that Bdf1 does not require Bdf2 to bind to its chromatin targets (Fig 6F). In addition, we were unable to detect any recruitment of Bdf2 to these genes, which raises the issue of the recruitment of Bdf2 to chromatin [55]. The fact that Bdf1 loading onto chromatin does not depend on Bdf2 reinforces the idea that Bdf2 does not contribute to the transcriptional regulation of middle sporulation genes.

## Discussion

In this study, the functional role of Bdf1, and more particularly its bromodomains, was analysed during sporulation. Our results showed that Bdf1, but not its bromodomains, is essential for growth on non-fermentable carbon sources. The bromodomains are, however, required for meiosis to progress and for mature spores to form. The gene expression program during sporulation when Bdf1 bromodomains are mutated is blocked in the transcriptional cascade at the point when Ndt80 intervenes, culminating in meiotic arrest and the non-expression of middle genes. Detailed characterisation of Bdf1 interactants revealed that it forms two exclusive complexes, one with Bdf2 and the other with the SWR complex. However, neither of these complexes is involved in the meiotic block observed when Bdf1 bromodomains are mutated. Therefore, we hypothesise that Bdf1 transcriptionally regulates meiotic genes without input from either of its major partners (Fig 5E).

### Bdf1 / Bdf2, carbon metabolism and sporulation

Here, and in previous studies, deletion of *BDF1* or *BDF2* was found to lead to severe growth defects in the absence of a fermentable carbon source [2,16,32]. Indeed, Bdf1 was shown to preserve mitochondrial activity in various metabolic conditions [6,56]. However, a putative role for Bdf2 has yet to be investigated. Our results indicated that this phenotype is not controlled by Bdf1 or Bdf2 bromodomains, thus possibly revealing a new function of BET proteins in cell metabolism, which is independent of their bromodomains and remains to be characterised.

Bdf1 was first linked to sporulation when its gene was cloned, its bromodomains were identified and the growth and sporulation defects induced by its deletion were analysed [16]. Furthermore, a transposon-based screen indicated that the second Bdf1 bromodomain, but not the first one, was essential for the completion of meiosis and spore formation [16]. Here, we found that both bromodomains of Bdf1 have to be mutated to impair the formation of spores and cause an arrest during meiosis. These apparent discrepancies compared to the results presented in Chua et al. [16] could be explained by the facts that (i) they used a transposon mutational screen which did not precisely disrupt Bdf1 bromodomains and (ii) a transposon insertion in the second bromodomain may have also disrupted the subsequent ET domain, which we also found to be essential for sporulation (Fig 6E, right).

When Bdf1 bromodomains are mutated, early inducers of the transcription program for sporulation are correctly expressed but transcriptional defects accumulate as sporulation progresses (Fig 3D, S2A Fig). In particular, expression of the middle sporulation genes and of the master regulator *NDT80* failed when Bdf1 bromodomains were not functional (Figs 4B and S2B). Interestingly, 65% of the genes downregulated in the bdf1 bromodomain mutant were also predicted targets of Ndt80 (Fig 4B). Several studies identified Bdf1 as important for gene splicing [15,34]. This functional role appears to be independent of its bromodomains as no splicing defects were observed in the *bdf1-Y187F-Y354F* mutant (S3D and S3E Fig).

### Bdf1 forms two exclusive complexes

Quantitative proteomics analysis of the Bdf1 interactome revealed that Bdf1’s main partners are Bdf2 and the SWR complex. These interactions occur as part of two different, mutually exclusive, complexes (Fig 5E). It is interesting to note that Bdf1, Bdf2 and Yaf9, the subunit of Swr1 which interacts with Bdf1, share similarities: first, they all contain domains which recognise acetylated lysines: bromodomains for Bdf1 and Bdf2 and a YEATS domain for Yaf9 [57]. The Yaf9 YEATS domain was recently described as a reader of lysine crotonylation and to promote active transcription [58]. Second, they all possess a coiled-coil domain, well known to mediate protein dimerization (S6 Fig, [59]). The B motif present in all the members of the BET family is also a coiled-coil domain and could mediate the dimerization of BET proteins [60]. Therefore, we hypothesised that coiled-coil domains could contribute to the formation of the two complexes the Bdf1 protein is involved in; Bdf1’s coiled-coil domain could interact exclusively either with the coiled-coil domain of Bdf2 or of Yaf9. This would explain how Bdf1 forms exclusive complexes with these two proteins.

In the SWR complex, Bdf1 promotes the incorporation of H2A.Z into acetylated regions [10,61,62]. The function of the interaction between Bdf1 and Bdf2 remains to be characterised. This interaction seems to be tight because deletion of any of the Bdf2 domains -bromodomains or ET- disrupts its interaction with Bdf1. However, none of these *BDF2* mutations induces any sporulation defects.

### Bdf1 is essential for the activation of meiotic-specific genes

Although Bdf1 and Bdf2 are functionally redundant—as only one is needed to maintain cell viability—they could play different roles in controlling chromatin dynamics. Indeed, they occupy distinct locations in the genome and larger amounts of Bdf1 bind to chromatin compared to Bdf2 [55]. Deletion of *BDF2* in vegetative cells only affects a few transcripts, whereas the deletion of *BDF1* provokes major transcriptomic changes [14]. The situation is similar during sporulation: Bdf1 is loaded on various loci, and especially middle sporulation genes, whereas Bdf2 does not seem to be strongly attached to chromatin (Fig 4F and ref [19]). The timing of the recruitment of Bdf1 onto chromatin is particularly intriguing, with Bdf1 detected on the promoters of middle sporulation genes before their transcriptional activation. This recruitment is essential for the activation of these genes and involves Bdf1 bromodomains. When these domains are mutated, Bdf1 is no longer recruited to these promoters and their genes fail to induce. The non-induction of *NDT80* causes the major defects observed when Bdf1 bromodomains are mutated. Indeed, this Bdf1 mutation phenocopies deletion of *NDT80*, which also induces stalling in the pachytene phase and failure to accumulate middle-meiotic mRNA. Moreover, the ectopic expression of *NDT80* can partially rescue the sporulation defects caused by mutation of Bdf1 bromodomains. However, the mechanisms through which Bdf1 specifically regulates the expression of *NDT80* and middle sporulation genes remain unclear. Bdf1 was originally described as a missing piece of the TFIID complex, although as a loosely interacting protein [63], which could partially explain why no TFIID proteins were identified in our purifications. Perhaps a minor fraction of Bdf1 interacts with the transcription machinery. Bdf1 alone could promote a chromatin state favourable for the expression of middle sporulation genes. Thus, Bdf1 could promote the initial low-level pool of Ndt80 in meiotic G2 and also promote the feed-forward autoregulatory loop required to trigger high expression levels of middle genes [64]. Bdf1 is a member of the BET family, which also includes the mammalian proteins Brd2, Brd3, Brd4 and Brdt. Brd4 has been shown to facilitate transcriptional activation and was recently found to have an intrinsic HAT activity [48]. However, the residues controlling this activity are not conserved in Bdf1. Finally, H4 acetylation and H4K5 butyrylation have been shown to regulate the binding of Brdt to chromatin, resulting in highly active transcription [65]. Similarly, different acyl marks could regulate Bdf1 binding to chromatin and may explain why the expression of middle genes specifically requires Bdf1 bromodomains.

This novel role for Bdf1 during yeast meiosis could be shared by mammalian BET proteins during spermatogenesis. Indeed, Brd2 and Brd4 are known to associate with mitotic chromosomes and Brd4 binds post-mitotic genes to promote their transcriptional reactivation after the M phase [66–68]. During male meiosis in mouse, Brdt is essential for the gene expression program, and its deletion affects the expression of testis-specific cyclin genes such as Ccna1, which are required for meiotic division [26]. In yeast, the results presented here revealed that mutation of Bdf1 bromodomains abolished the expression of almost all the subunits of the APC complex, which is required for meiotic progression [69]. Interestingly, in mice, the absence of Brdt leads to a loss of *FZR1* expression—an essential activator of the APC complex during meiosis—and subsequent deregulation of the APC complex (S7 Fig and reference [70]).

In conclusion, Bdf1 appears to be a key chromatin protein for the meiotic transcription program, regulating the expression of middle sporulation genes and promoting their rapid activation. Its role during meiosis appears to be evolutionarily conserved from yeast to mouse, and BET proteins are essential for the functionality of the APC complex and its regulation of meiotic progression. Our results suggest that the control of gene activation by Bdf1 does not rely on any of its major protein partners. Further studies will be required to explore its precise mode of action and whether these molecular mechanisms are conserved during the mammalian cell cycle in somatic cells and during meiosis and gamete differentiation.

## Materials and Methods

### Antibodies

GST, TAP and HA antibodies were obtained from Dutscher (Ref. 27-4577-01), Fisher (Ref. 10506450) and Roche (Ref. 11867423001), respectively. Anti-Bdf1 antibody was developed in-house by injecting recombinant protein into rabbits (Covalab). The use of *bdf1*Δ and Bdf1-TAP strains validated its specificity (S4A Fig).

### Cloning, expression, and purification of proteins

Yeast Bdf1 bromodomain 1 (residues 132–263) or 2 (residues 317–430) and human Brd4 bromodomain 1 (residues 22–204) were cloned into pGEX-4T1 as GST-tagged proteins. Expression was induced in *E*. *coli* strain BL21 (DE3) grown in LB medium with kanamycin (50 μg/mL) at 37°C by adding 0.5 mM IPTG at OD_600_ of 1 and then incubated for 16 h at 16°C. Cells were lysed by sonication in Tris-HCl 50 mM pH 7.5, NaCl 150 mM and protease inhibitors. Clarified lysate was incubated with glutathione sepharose (Fisher, ref. W7349W) and washed in Tris-HCl 50 mM pH 7.5, NaCl 500 mM, NP-40 1%. Bound proteins were eluted in glutathione 10 mM.

### Histone tail peptides arrays and peptide pull-down

For peptide pull-downs, 0.5 nmol of biotinylated H4 (Millipore, ref. 12–405) and H4K5ac K8ac K12ac K16ac peptides (H4ac4, H4 (Millipore, ref. 12–379)) were bound to Streptavidin magnetic beads (Thermo Dynabeads MyOne Streptavidin, ref. 65001) and used to pull-down 1.25 μg of GST-tagged Bdf1 bromodomain 1 and 2 (wild-type and mutants, *bdf1-Y187F* and *bdf1-Y354F*) and GST-tagged Brd4 bromodomain 1 of Brd4 in Tris-HCl pH 7.5 50 mM, NaCl 150 mM, NP-40 0.1%, Glycerol 10% and DTT 1 mM. Washes were optimized using 500 mM of NaCl. Bound proteins were eluted by boiling in Leammli buffer and proteins analysed by western blot using an anti-GST antibody.

Histone peptide array were purchased from Active Motif (ref. 13005) and used in accordance with the supplier’s recommendations. Briefly, arrays were blocked and then incubated with 1 μM of purified GST-tagged Bdf1 bromodomain 1 and 2. Incubation was allowed to proceed for 2 h at 4°C in binding buffer (Tris-HCl 50 mM pH 7.4, NP-40 0.1%, NaCl 150 mM, glycerol 10%). The membrane was washed in PBS Tween 0.1% and detected with an anti-GST antibody mixed with an anti-myc antibody used as a positive control and for normalisation. The membrane was washed and incubated with horseradish-peroxidase-conjugated anti-Goat antibody (Jackson, ref. 705.035.147). The membrane was submerged in Clarity Western ECL Substrate (Bio-Rad, ref. 170–5060), imaged (Bio-Rad ChemiDoc XRS Imaging) and data were quantified using an array analyser software provided by Active Motif.

Signal intensities are presented in S1 Table.

### Yeast strains, DNA recombinant work, and microbiological techniques

Yeast strains, plasmids and primers are listed in S2, S3 and S4 Tables respectively. The genotypes/expression profiles of all deletion mutants and tagged strains were confirmed by PCR analysis, sequencing and/or western blot analysis. Frogging assays on agar plates used 10-fold serial dilutions.

### Sporulation

Diploid yeast in the SK1 background was grown to an OD_600_ of 0.5 in YPD. Cells were washed and resuspended in YPA at an OD_600_ of 0.03. After 12 h cells were washed and transferred into sporulation media (K acetate 2%) supplemented with auxotrophic amino acids. Six hours after sporulation induction, *GAL-NDT80 GAL4*.*ER* strain was released from the meiotic arrest by the addition of 1 μM oestradiol (5 mM stock in ethanol, Sigma E2758-1G). Sporulation efficiency was assessed after 24 h of induction and is defined by the number of cells which formed tetrads.

### DAPI staining

Meiotic progression was monitored by fixing 500 μl of cells in EtOH 70% and staining the nuclei with 0.5 μg/ml of DAPI. Fluorescence microscopy was used to determine the proportion of mono-, bi-, tri- and tetra-nucleated cells.

### Meiotic recombination analysis

The frequency of meiotic recombination between the heteroalleles *his4-N/his4-G* was quantified by plating sporulated yeast cultures on YPD and SC-HIS plates. The number of yeasts growing on SC-HIS plates was counted and normalised to the total number of cells growing on YPD.

### RNA-seq and bioinformatics analysis

Samples were collected from sporulating cells (wild-type and Bdf1 double point mutant *bdf1-Y187F-Y354F*) at 0, 4 and 8 h. Cell pellets were washed and stored at -80°C. Total RNA was extracted with phenol:chloroform (Sigma Ref. 2190191) from three independent biological replicates. DNaseI treatment (Thermo, ref. AM2222) was performed using 1 μg of total RNA subsequently purified by phenol:chloroform extraction. Intact poly(A)+ RNA was isolated by using the NEB Next Poly(A)+ mRNA Magnetic Isolation kit (E7490S). cDNA libraries were constructed from 10 ng of total RNA using the NEB Next Ultra RNA Library Prep Kit for Illumina (New England Biolabs, ref. E7530S). Fragments were enriched by 15 cycles of PCR amplification. Agilent 2100 Bioanalyzer (2100, Agilent Technologies, CA) was used to assess the quality and quantity of each library. Eighteen barcoded cDNA libraries were pooled and sequenced on an Illumina HiSeqTM 2000, generating 5–8 million single reads (SR50) for each sample. The bioinformatic analysis pipeline, presented S3A Fig can be summarized as follows: reads were quality-checked using FastQC before and after cleaning by Trimmomatic applying default parameters [71]. Reads were then aligned to *S*. *cerevisiae* S288c genome build R64-1-1.82 using Bowtie2 [72]. The attribute file was downloaded from Ensembl website and modified to include yeast intronic sequences, obtained from Yeastmine. Raw read counts for each gene were calculated using HTSeqCount using defaults parameters [73]. Read count data was normalised using the DESeq2 R-package (S5 Table, [74]). DESeq2 normalised read counts were used to identify differentially expressed genes with an adjusted p-value < 0.05 and a fold change below -2 or above 2 (S5 Table). Gene ontology analysis was performed using the generic GO-term mapper developed by the Max Planck Institute (http://cpdb.molgen.mpg.de/YCPDB).

Pscan tool was used to explore the promoter of the genes which are downregulated in *bdf1-Y187F-Y354F* 4 and 8h after sporulation induction (http://159.149.160.88/pscan/, [75]). Binding motifs for yeast transcription factors were obtained from the JASPAR 2016 database [76] and researched in the promoter of each gene (-500 to 0 bp upstream each transcription start side). A binding motif was considered significantly over-represented when the p-value was inferior to 0.001 (Fig 4A).

The probability that an Ndt80 binding sequence was present in the promoter of *bdf1-Y187F-Y354F* deregulated genes was calculated using the Morpheus ROC tool [43]. This tool computes the ROC-AUC value as a measure of the enrichment of the binding sequence of a transcription factor in a list of candidate genes compared to a mock list of genes (Fig 4C).

Venn diagram was generated using Venny 2.1–0 (http://bioinfogp.cnb.csic.es/tools/venny/index.html). A list of genes potentially regulated by Ndt80 was obtained from Chu et al [35]. The statistic representation of these genes in the list of genes downregulated in the *bdf1-Y187F-Y354F* mutant was tested using a hypergeometric test (Fig 4D).

### Chromatin immunoprecipitation

ChIP analyses were performed as described [19] with minor changes. Crosslinking was done with 1% EGS for 15 min and then 1% formaldehyde for 10 min before quenching for 5 min with Glycine 125 mM. Cells were lysed in lysis buffer (Hepes 50 mM pH 7.5, NaCl 140 mM, EDTA 1mM, Triton X-100 0.1%, PMSF 0.5 mM, cOmplete, TSA 100 mg/l, phosphatase inhibitor cocktail (Sigma, ref. P2850)) in a Fastprep (MP Biologicals) for three periods of 45 s. Extracts were sonicated in cycles of 20 s with intermediate incubation for 40 s over a total of 30 min (EpiShear, Active Motif). Clarified extracts were immunoprecipitated by incubating with Pan-Mouse IgG Dynabeads (LifeTechnologies, ref. 11041) or with Protein G Dynabeads (Thermo, ref. 10004D) conjugated with anti-HA antibody (Roche, ref. 11867423001) for TAP or HA tagged strains, respectively. ChIP data are presented as percent of input. In all cases, at least three biological replicates were performed to determine the standard errors in each experiment.

### Tandem affinity purification and interactomic analyses

TAP purifications were performed as described previously [77,78]. Calmodulin eluates from the TAP-purified complexes were analysed by SDS-PAGE by using Novex 4–12% gradient gels (Invitrogen) and visualised by staining with SilverQuest Silver Staining Kit (Thermo, LC6070). Western blot analysis of the calmodulin eluates was performed using the corresponding antibodies. Protein preparation and mass spectrometry-based proteomic analyses were carried out as described in [79]. Briefly, eluted proteins were stacked as a single band in a SDS-PAGE gel (NuPAGE 4–12%, Invitrogen) and submitted to in-gel digestion using trypsin (Promega, sequencing grade). Resulting peptides were analysed by online nanoLC-MS/MS (UltiMate 3000 and Q-Exactive Plus or LTQ-Orbitrap Velos Pro, Thermo Scientific) using a 120-min gradient. Peptides and proteins were identified and quantified using MaxQuant (version 1.5.3.30, [80]) and the SGD database (November 2015 release). Proteins were quantified based on the iBAQ value [81] calculated by MaxQuant. Only proteins identified with a minimum of two unique + razor peptides were taken into account. For statistical analysis of results obtained with Bdf1-TAP triplicates, we used ProStaR [82]: proteins identified in the reverse and contaminant databases and proteins exhibiting fewer than 6 iBAQ values in a single condition (2 conditions and 2 analytical replicates per biological replicate) were discarded from the list. After log_2_ transformation, iBAQ values were normalised by condition-wise median centring before imputing missing values using the QRILC algorithm (missing not at random-devoted imputation method); statistical testing was conducted using *limma*. For a protein to be considered as a potential binding partner of Bdf1-TAP, it had to exhibit a p-value < 0.001 and a log_2_ (fold change) ≥ 7.

### Coimmunoprecipitation

Coimmunoprecipitation was performed using 50 mL of cells grown on YPD to an OD_600_ of 0.5. Cells were harvested, washed with water, and resuspended in lysis buffer (HEPES 50 mM pH 7.5, NaCl 140 mM, EDTA 1 mM, glycerol 10%, NP-40 0.5%, PMSF 1 mM and protease inhibitors). An equal volume of glass beads was added. Cells were lysed in a Fastprep homogenizer (MP Biomedicals) for 45 s. The clarified extracts were immunoprecipitated during 2 h at 4°C using Dynabeads Pan Mouse IgG (LifeTechnologies). The immunoprecipitates were washed and resuspended in 50 μl of SDS-PAGE sample buffer for subsequent western blot analysis.

### RT-qPCR

Reverse transcription was performed by following manufacturer instructions using kits iScript RT Supermix and Universal SYBR Green Supermix (Bio-Rad) on a CFX384 Touch qPCR machine (Bio-Rad). Primers are presented (S4 Table). At least three biological replicates were performed to determine the standard errors for each experiment. RT-qPCR data were then normalised relative to the reference gene, *NUP85* [19].

### Accession numbers

RNA-seq data obtained in this study are available from the GEO repository (GSE89530). Mouse transcriptomic data was derived from RNA-seq dataset GEO GSE39909 as described in (S7 Fig, [26]). Proteomic data produced in this study have been deposited on ProteomeXchange under identifier PXD005227.

## Supporting Information

S1 Fig Additional information on BET bromodomains and Bdf1 affinity for acetylated H4 peptides. (A) Sequence alignments of human BET and Sc-Bdf1 bromodomains. Bdf1 residues mutated in this study are highlighting. (B) Pull-down assay using histone H4 (H4) and tetra-acetylated H4 peptides (H4K5ac K8ac K12ac K16ac, H4ac4) on human Brd4-BD1, Bdf1-BD2 and Bdf1-BD2-Y338W proteins.
(TIF)

S2 Fig RT-qPCR profile of representative genes illustrating the progression of sporulation in WT and *bdf1-Y187F-Y354F* strains. Early, middle and late sporulation genes are shown in (A), (B) and (C), respectively.
(TIF)

S3 Fig Additional information on the transcriptomic analysis in the *bdf1-Y187F-Y354F* strain during sporulation. (A) Pipeline for the bioinformatic analysis of the RNA-seq data. QC, Quality Check. (B) Top, Expression levels for *BDF1* and *BDF2* during sporulation, expressed in normalised counts. Bottom, Bdf1 expression levels in WT and *bdf1-Y187F-Y354F* strains analysed by western blot. (C). Principal Component Analysis applied on the normalized counts of each sample. Principal components are represented on the x- and y-axis. Data for WT cells are shown in black; data for *bdf1-Y187F-Y354F* strains are represented in red. Sporulation progression is identified by the following shapes: 0 h, round; 4 h, triangle; 8 h, square. All three replicates are presented and are sometimes superposed and indistinguishable. (D) Normalized read counts present in introns, expressed as the log2 of the ratio *bdf1-Y187F-Y354F* vs WT. Introns from five representative reporters genes are presented, as well as the average ratio for all introns [15]. (E) Normalized counts observed in the *AMA1* intron during sporulation in the WT and *bdf1-Y187F-Y354F* strains. (F) GO term enrichment analysis using differentially expressed genes in Bdf1 bromodomain mutants. The proportion of differentially expressed genes within the indicated GO term is indicated on the x-axis.
(TIF)

S4 Fig Specificity of the Bdf1 antibody and confirmation of the interaction of Bdf1 with Bdf2 and Swr1. (A) Specificity of the Bdf1 antibody. It was used to detect Bdf1 in WT, *bdf1*Δ and *BDF1-TAP* strains. Star (*) indicates a non-specific band. (B) Bdf1 was identified by western blot in the protein eluates after TAP purification from TAP-tagged Bdf2 and Swr1 strains.
(TIF)

S5 Fig Additional phenotypic testing on Bdf1 strains. (A) Sporulation efficiency of *BDF1* tagged strains with HA or TAP tags. (B) Growth assay of *bdf1-ETΔ* mutants on fermentable (glucose, YPD) and non-fermentable (acetate, YPA and glycerol, YPG) carbon sources.
(TIF)

S6 Fig Coiled-coil dimerization domain might control Bdf1 interaction with Bdf2 or Yaf9. (A) Localization of a coiled-coil domain in Bdf1, Bdf2 and Yaf9. (B) Schematization of amino acid organisation in a coiled-coil dimerization (top). Alignments of Bdf1, Bdf2 and Yaf9 with human BET coiled-coil domain indicate the conservation of hydrophobic (orange) and charged amino acids (purple) which are important for the formation of coiled-coil domains [83].
(TIF)

S7 Fig Analysis of *BRDT* and *FZR1* expression levels in testis from Brdt knock out mice. Data were generated from GEO dataset GSE39909 [26].
(TIF)

S1 Table Summary of histone peptide array data. (XLSX)

S2 Table List of yeast strains. (PDF)

S3 Table List of plasmids. (PDF)

S4 Table List of primers. (PDF)

S5 Table Additional information of the transcriptomic analysis of WT and *bdf1-Y187F-Y354F* strains during sporulation. This table contains the normalised counts for each gene in each condition, the list of differentially expressed genes and the list of genes with an Ndt80 binding sequence in their promoters. Datasets have been deposited in GEO under accession number GSE89530.
(XLSX)

S6 Table List of specific partners identified and quantified by mass spectrometry (Bdf1, Bdf2 and Swr1 purifications). (XLSX)
