# Supplementary figures and images for: Bdf1 Bromodomains Are Essential for Meiosis and the Expression of Meiotic-Specific Genes
Source: PLoS Genet. 2017 Jan 9;13(1):e1006541. doi: 10.1371/journal.pgen.1006541 (PMC5261807; doi:10.1371/journal.pgen.1006541)

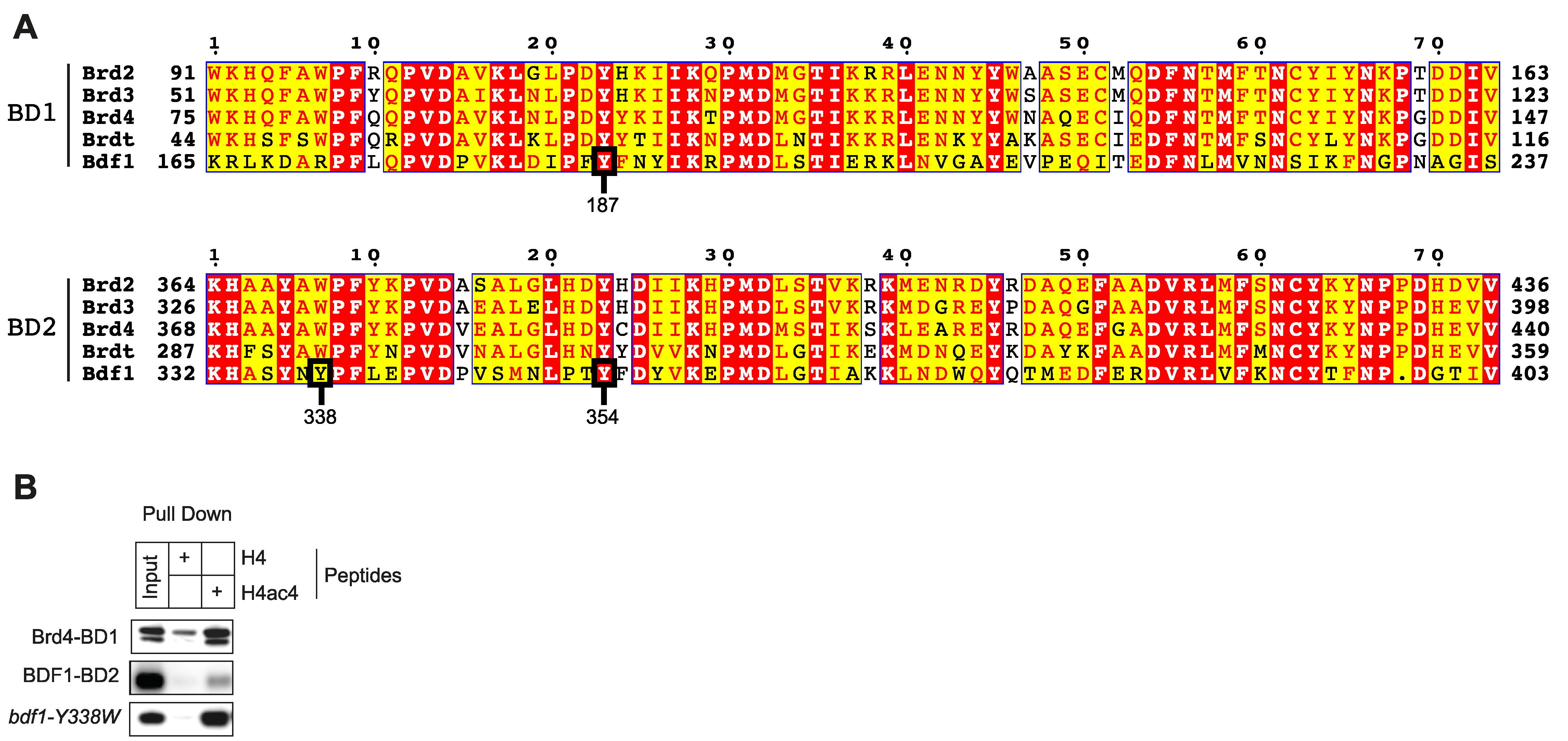

Supplement: S1 Fig — (A) Sequence alignments of human BET and Sc-Bdf1 bromodomains. Bdf1 residues mutated in this study are highlighting. (B) Pull-down assay using histone H4 (H4) and tetra-acetylated H4 peptides (H4K5ac K8ac K12ac K16ac, H4ac4) on human Brd4-BD1, Bdf1-BD2 and Bdf1-BD2-Y338W proteins. (TIF) [file pgen.1006541.s001.tif]

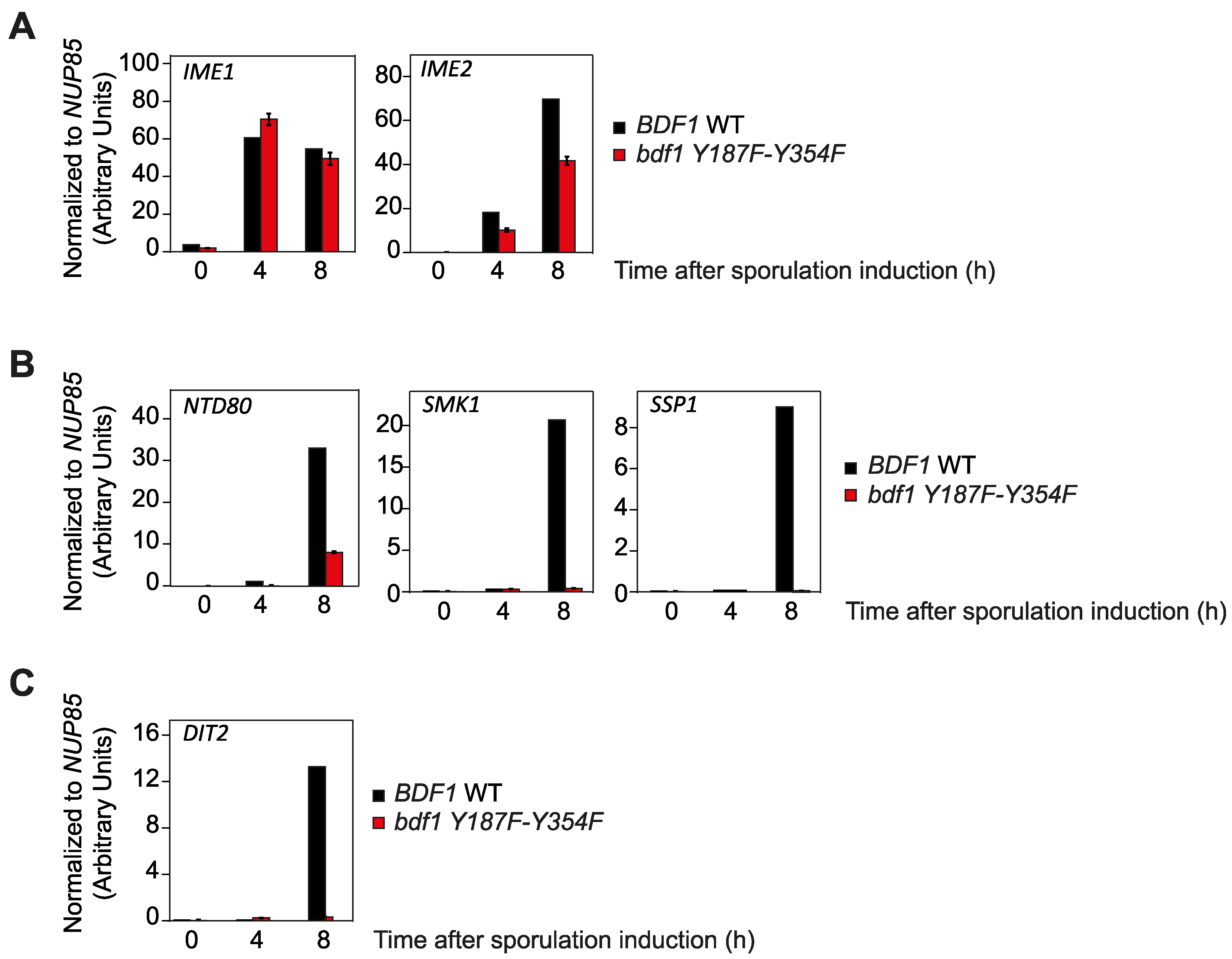

Supplement: S2 Fig — Early, middle and late sporulation genes are shown in (A), (B) and (C), respectively. (TIF) [file pgen.1006541.s002.tif]

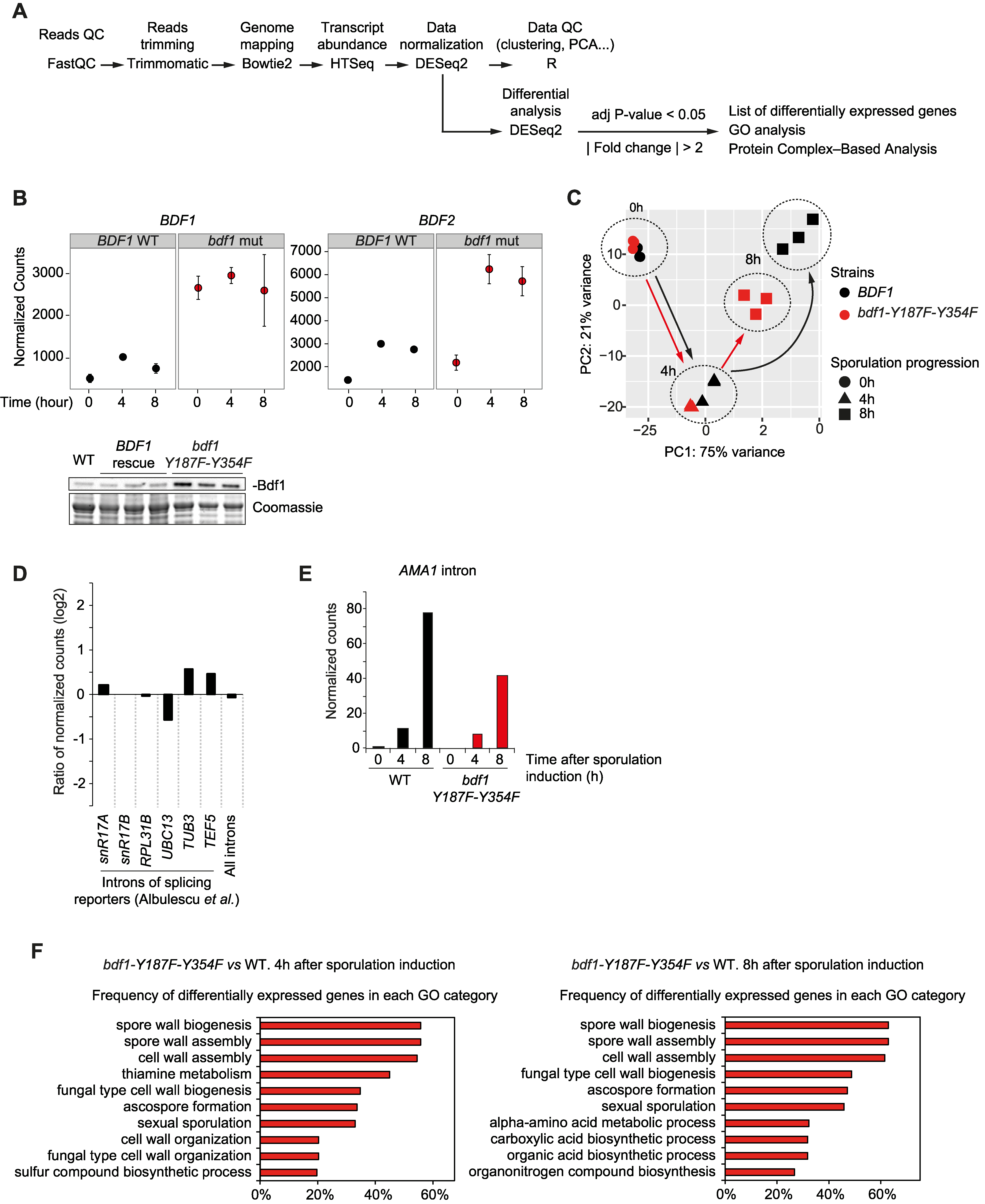

Supplement: S3 Fig — (A) Pipeline for the bioinformatic analysis of the RNA-seq data. QC, Quality Check. (B) Top, Expression levels for BDF1 and BDF2 during sporulation, expressed in normalised counts. Bottom, Bdf1 expression levels in WT and bdf1-Y187F-Y354F strains analysed by western blot. (C). Principal Component Analysis applied on the normalized counts of each sample. Principal components are represented on the x- and y-axis. Data for WT cells are shown in black; data for bdf1-Y187F-Y354F strains are represented in red. Sporulation progression is identified by the following shapes: 0 h, round; 4 h, triangle; 8 h, square. All three replicates are presented and are sometimes superposed and indistinguishable. (D) Normalized read counts present in introns, expressed as the log2 of the ratio bdf1-Y187F-Y354F vs WT. Introns from five representative reporters genes are presented, as well as the average ratio for all introns [15]. (E) Normalized counts observed in the AMA1 intron during sporulation in the WT and bdf1-Y187F-Y354F strains. (F) GO term enrichment analysis using differentially expressed genes in Bdf1 bromodomain mutants. The proportion of differentially expressed genes within the indicated GO term is indicated on the x-axis. (TIF) [file pgen.1006541.s003.tif]

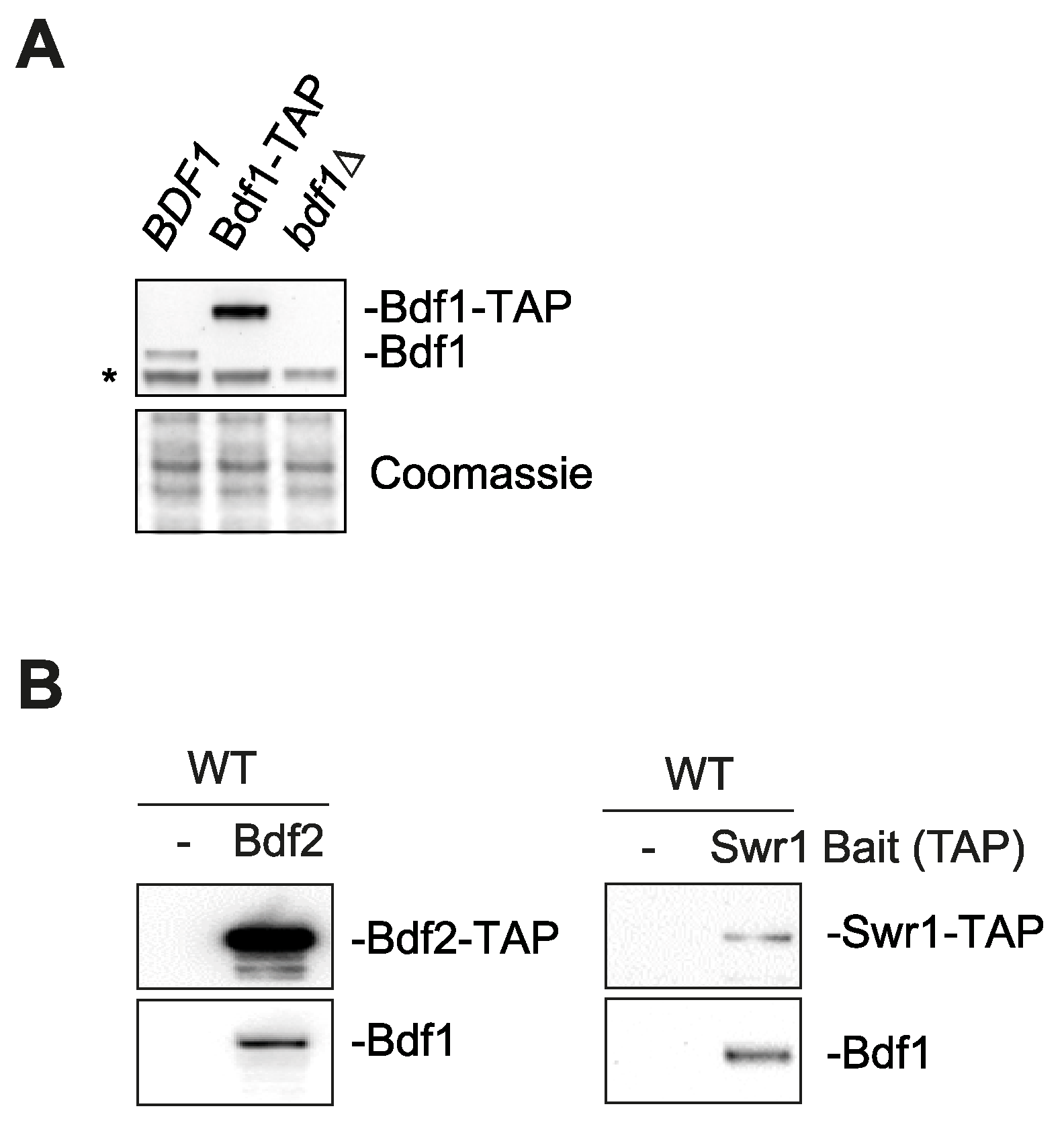

Supplement: S4 Fig — (A) Specificity of the Bdf1 antibody. It was used to detect Bdf1 in WT, bdf1Δ and BDF1-TAP strains. Star (*) indicates a non-specific band. (B) Bdf1 was identified by western blot in the protein eluates after TAP purification from TAP-tagged Bdf2 and Swr1 strains. (TIF) [file pgen.1006541.s004.tif]

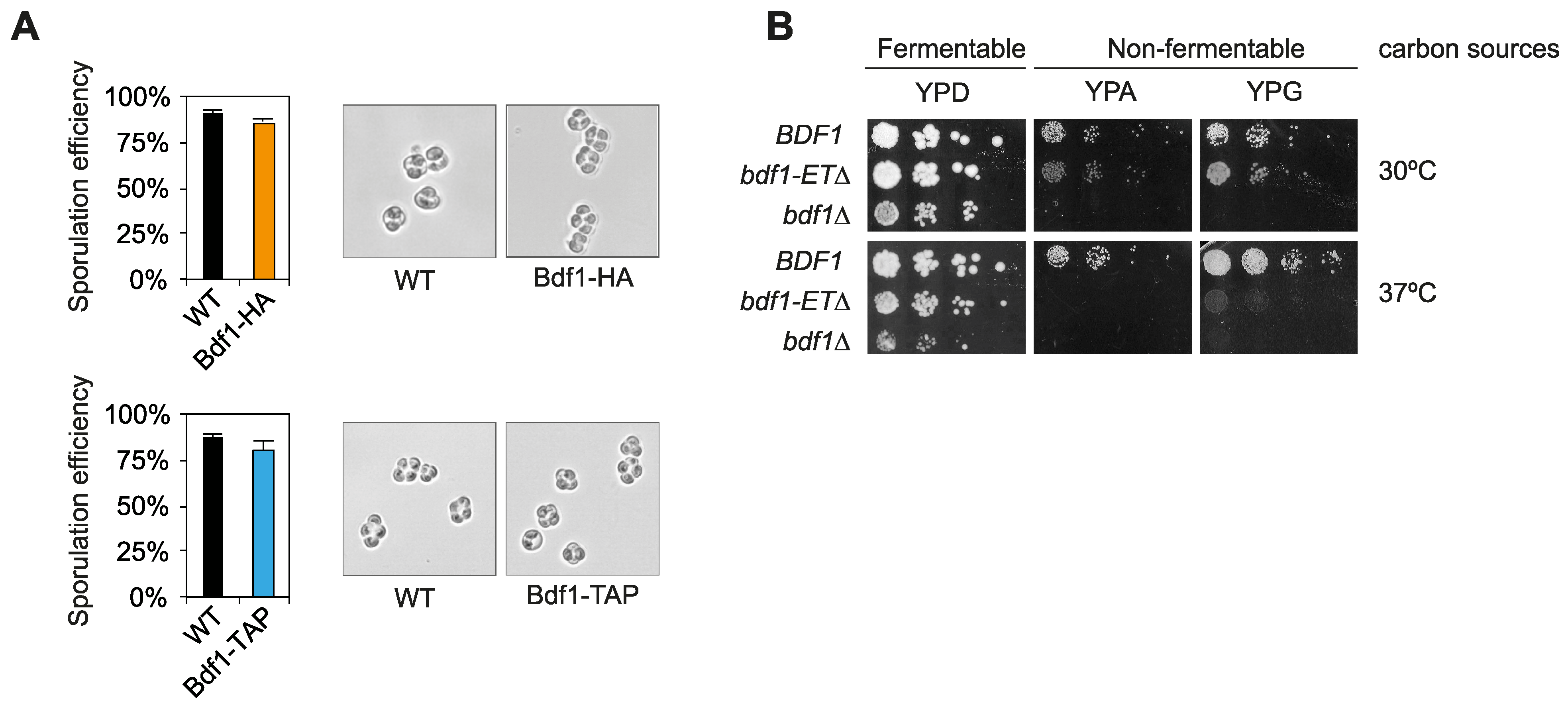

Supplement: S5 Fig — (A) Sporulation efficiency of BDF1 tagged strains with HA or TAP tags. (B) Growth assay of bdf1-ETΔ mutants on fermentable (glucose, YPD) and non-fermentable (acetate, YPA and glycerol, YPG) carbon sources. (TIF) [file pgen.1006541.s005.tif]

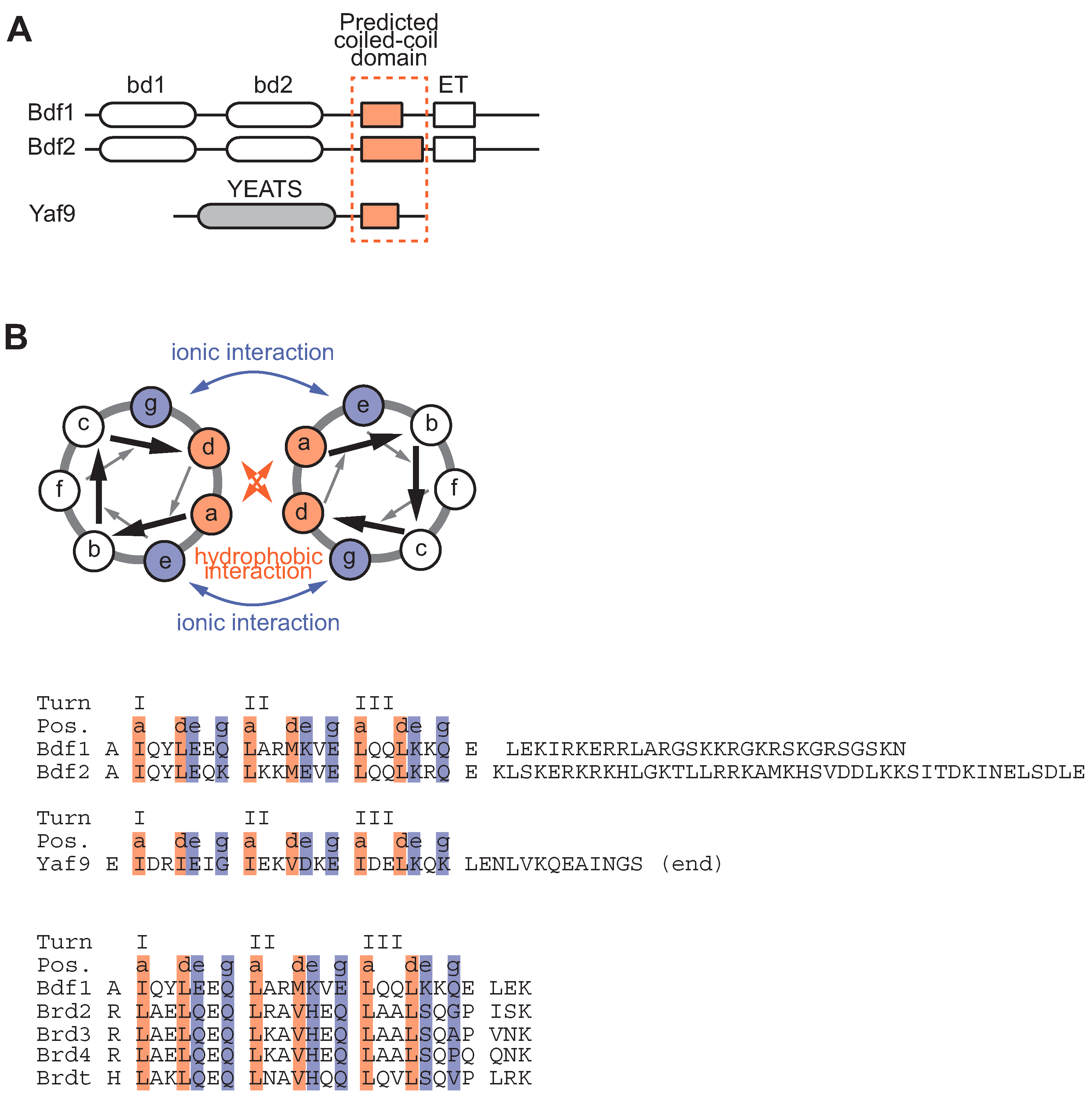

Supplement: S6 Fig — (A) Localization of a coiled-coil domain in Bdf1, Bdf2 and Yaf9. (B) Schematization of amino acid organisation in a coiled-coil dimerization (top). Alignments of Bdf1, Bdf2 and Yaf9 with human BET coiled-coil domain indicate the conservation of hydrophobic (orange) and charged amino acids (purple) which are important for the formation of coiled-coil domains [83]. (TIF) [file pgen.1006541.s006.tif]

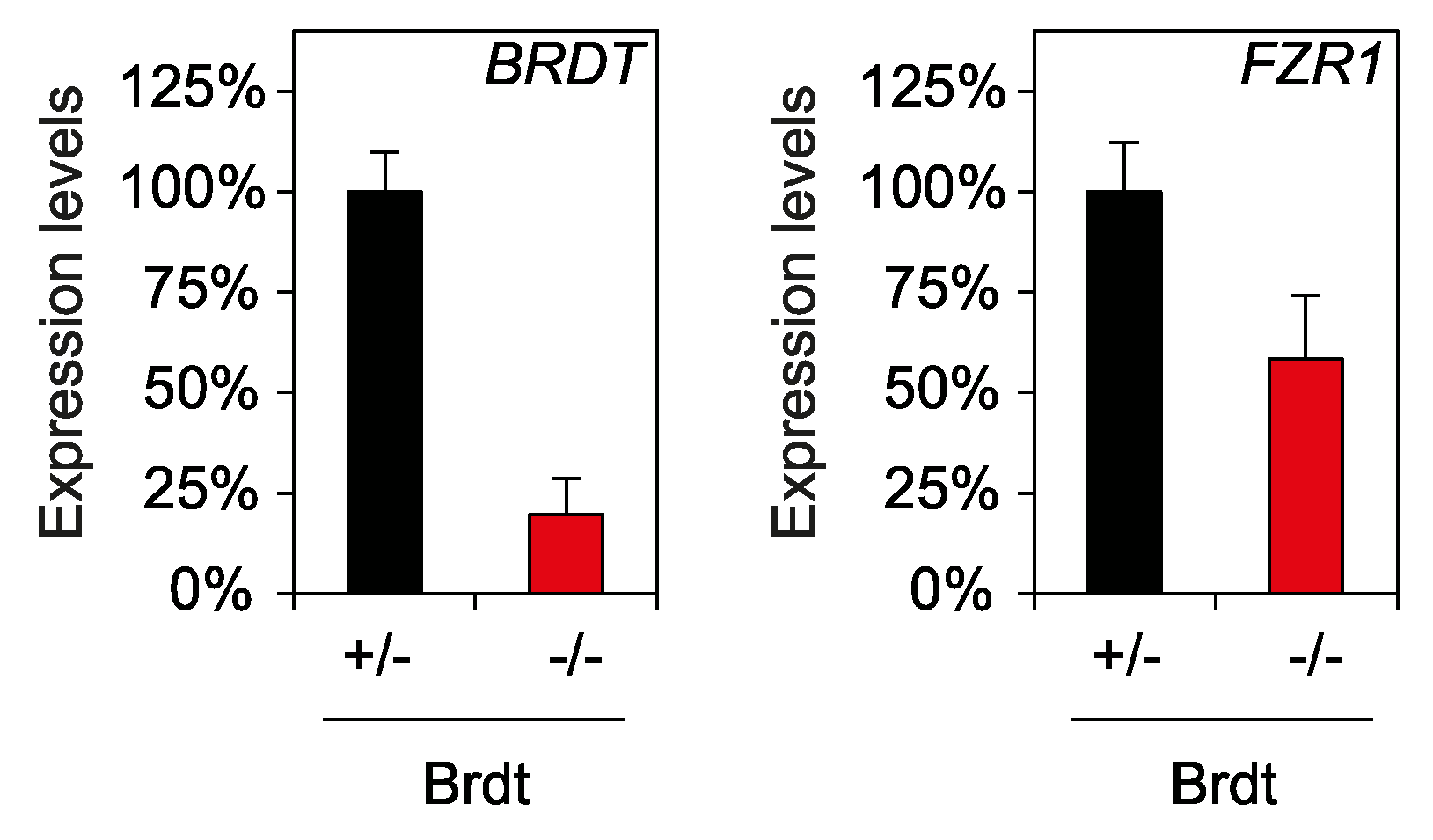

Supplement: S7 Fig — Data were generated from GEO dataset GSE39909 [26]. (TIF) [file pgen.1006541.s007.tif]
