# Supplementary material for: Bdf1 Bromodomains Are Essential for Meiosis and the Expression of Meiotic-Specific Genes
Source: PLoS Genet. 2017 Jan 9;13(1):e1006541. doi: 10.1371/journal.pgen.1006541 (PMC5261807; doi:10.1371/journal.pgen.1006541)
Supplement: S2 Table — (PDF) [file pgen.1006541.s009.pdf]

Table S2. List of yeast strains. They are listed by order of appearance in the manuscript.

Name	Description	Genotype	Background	Source
yJG373	*BDF1::TRP1*	MATa/alpha leu2::hisG/" trp1::hisG/" lys2-SK1/" his4- N/his4-G ura3-SK1/" ho::*LYS2*/" *BDF1::TRP1*/"	SK1	This study
yJG387	*bdf1-bd1Δ*	MATa/alpha leu2::hisG/" trp1::hisG/" lys2-SK1/" his4- N/his4-G ura3-SK1/" ho::*LYS2*/" *bdf1-bd1Δ*::*TRP1*(591-827 deleted)/"	SK1	This study
yJG389	*bdf1-bd2Δ*	MATa/alpha leu2::hisG/" trp1::hisG/" lys2-SK1/" his4- N/his4-G ura3-SK1/" ho::*LYS2*/" *bdf1-bd2Δ*::TRP1(855-1091 deleted)/"	SK1	This study
yJG372	*bdf1-bd1Δ-bd2Δ*	MATa/alpha leu2::hisG/" trp1::hisG/" lys2-SK1/" his4- N/his4-G ura3-SK1/" ho::*LYS2*/" *bdf1-bd1Δ-bd2Δ*::*TRP1*(591-827, 855-1091 deleted)/"	SK1	This study
yJG341	*bdf1-Y187F*	MATa/alpha leu2::hisG/" trp1::hisG/" lys2-SK1/" his4- N/his4-G ura3-SK1/" ho::*LYS2*/" *bdf1-Y187F*::TRP1/"	SK1	This study
yJG342	*bdf1-Y354F*	MATa/alpha leu2::hisG/" trp1::hisG/" lys2-SK1/" his4- N/his4-G ura3-SK1/" ho::*LYS2*/" *bdf1-Y354F*::TRP1/"	SK1	This study
yJG349	*bdf1-Y187F-Y354F*	MATa/alpha leu2::hisG/" trp1::hisG/" lys2-SK1/" his4- N/his4-G ura3-SK1/" ho::*LYS2*/" *bdf1-Y187F-Y354F*::*TRP1*/"	SK1	This study
yJG178	WT	MATa ura3 leu2::hisG trp1::hisG lys2 ho::LYS2 met4-445 *his3Δ*	SK1	Ed Winter
yJG302	Bdf1-TAP	MATa ura3 leu2::hisG trp1::hisG lys2 ho::LYS2 met4-445 *his3Δ BDF1*-TAP*::HIS3MX6*	SK1	This study
yJG306	Bdf2-TAP	MATa ura3 leu2::hisG trp1::hisG lys2 ho::LYS2 met4-445 *his3Δ BDF2*-TAP*::HIS3MX6*	SK1	This study
yJG360	Bdf1-TAP *bdf2Δ*	MATa ura3 leu2::hisG trp1::hisG lys2 ho::LYS2 met4-445 *his3Δ BDF1*-TAP*::HIS3MX6 bdf2Δ::KANMX4*	SK1	This study
yJG408	Bdf2-TAP *bdf1Δ*	MATa ura3-SK1 leu2::hisG trp1::hisG lys2 ho::LYS2 met4-445 *his3Δ BDF2-*TAP*::HIS3MX6 bdf1Δ::KANMX4*	SK1	This study
yJG122	WT	MATa his3Δ1 leu2Δ0 met15Δ0 ura3Δ0	BY4741 (s288c)	Open Biosystems
yJG300	Bdf1-TAP	MATa his3Δ1 leu2Δ0 met15Δ0 ura3Δ0 *BDF1*-TAP::*HIS3MX6*	BY4741	Open Biosystems
yJG407	Swr1-TAP	MATa his3Δ1 leu2Δ0 met15Δ0 ura3Δ0 *SWR1*-TAP::*HIS3MX6*	BY4741	Open Biosystems
yJG336	Bdf1-TAP *yaf9Δ*	MATa his3Δ1 leu2Δ0 met15Δ0 ura3Δ0 *BDF1*-TAP::HIS3MX6 *yaf9*::*KANMX4*	BY4741	This study
yJG156	*bdf1Δ*	MATa/alpha leu2::hisG/" trp1::hisG/" lys2-SK1/" his4- N/his4-G ura3-SK1/" ho::*LYS2*/" *bdf1::KANMX4/"*	SK1	This study
yJG391	*bdf2Δ*	MATa/alpha leu2::hisG/" trp1::hisG/" lys2-SK1/" his4- N/his4-G ura3-SK1/" ho::*LYS2*/" *bdf2::KANMX4/"*	SK1	This study

yJG426	*yaf9Δ*	MATa/alpha leu2::hisG/" trp1::hisG/" lys2-SK1/" his4- N/his4-G ura3-SK1/" ho::*LYS2*/" *yaf9::KANMX4*	SK1	This study
yJG427	*swr1Δ*	MATa/alpha leu2::hisG/" trp1::hisG/" lys2-SK1/" his4- N/his4-G ura3-SK1/" ho::*LYS2*/" *swr1::KANMX4*	SK1	This study
yJG441	*bdf2-bd1Δ-bd2Δ-TAP*	MATa/alpha ura3-SK1/" leu2::hisG/" trp1::hisG/" lys2/" ho::LYS2/" met4-445/" *his3Δ/" bdf2-BD1Δ-BD2Δ*-TAP::*HIS3/"*	SK1	This study
yJG442	*bdf2-ETΔ-TAP*	MATa/alpha ura3-SK1/" leu2::hisG/" trp1::hisG/" lys2/" ho::LYS2/" met4-445/" *his3Δ/" bdf2-ETΔ-TAP*::*HIS3*	SK1	This study
yJG438	*bdf1-ETΔ*	MATa/alpha leu2::hisG/" trp1::hisG/" lys2-SK1/" his4- N/his4-G ura3-SK1/" ho::*LYS2*/" *bdf1-ETΔ::TRP1*	SK1	This study
yJG529	ER *bdf1-Y187F-Y535F*	MATa/alpha leu2::hisG/" trp1::hisG/" lys2-SK1/" his4 ura3-SK1/" ho::*LYS2*/" pGPD1-*GAL4*(848).ER::*URA3/"* GAL-*NDT80::TRP1/" bdf1-Y187F-Y535F::TRP1/"*	SK1	This study
yJG530	ER *BDF1::TRP1*	MATa/alpha leu2::hisG/" trp1::hisG/" lys2-SK1/" his4 ura3-SK1/" ho::*LYS2*/" pGPD1-*GAL4*(848).ER::*URA3/"* GAL-*NDT80::TRP1/" BDF1::TRP1/"*	SK1	This study
yJG535	*bdf1-Y187F-Y354F*-HA	MATa/alpha leu2::hisG/" trp1::hisG/" lys2-SK1/" his4- N/his4-G ura3-SK1/" ho::*LYS2*/" *bdf1-Y187F-Y354F*-HA::*KANMX4*/"	SK1	This study
yJG536	Bdf1-HA	MATa/alpha leu2::hisG/" trp1::hisG/" lys2-SK1/" his4- N/his4-G ura3-SK1/" ho::*LYS2*/" *BDF1-HA*::*KANMX4*/"	SK1	This study
yJG521	*BDF1::TRP1 sum1Δ*	MATa/alpha leu2::hisG/" trp1::hisG/" lys2-SK1/" his4- N/his4-G ura3-SK1/" ho::*LYS2*/" *sum1Δ::KANMX4/ BDF1::TRP1/"*	SK1	This study
yJG522	*bdf1-Y187F-Y354F sum1Δ*	MATa/alpha leu2::hisG/" trp1::hisG/" lys2-SK1/" his4- N/his4-G ura3-SK1/" ho::*LYS2*/" *sum1Δ::KANMX4/" bdf1-Y187F-Y354F::TRP1/"*	SK1	This study
yJG523	*BDF1::TRP1 hst1Δ*	MATa/alpha leu2::hisG/" trp1::hisG/" lys2-SK1/" his4- N/his4-G ura3-SK1/" ho::*LYS2*/" *hst1Δ::KANMX4/" BDF1::TRP1/"*	SK1	This study
yJG524	*bdf1-Y187F-Y354F hst1Δ*	MATa/alpha leu2::hisG/" trp1::hisG/" lys2-SK1/" his4- N/his4-G ura3-SK1/" ho::*LYS2*/" *hst1Δ::KANMX4/" bdf1-Y187F-Y354F::TRP1/"*	SK1	This study
yJG493	*sum1Δ*	MATa/alpha leu2::hisG/" trp1::hisG/" lys2-SK1/" his4- N/his4-G ura3-SK1/" ho::*LYS2*/" *sum1Δ::KANMX4/"*	SK1	Ed Winter (ALY50)
yJG496	*hst1Δ*	MATa/alpha leu2::hisG/" trp1::hisG/" lys2-SK1/" his4- N/his4-G ura3-SK1/" ho::*LYS2*/" *hst1Δ::KANMX4/"*	SK1	Ed Winter (ALY27)
