# Supplementary material for: Bdf1 Bromodomains Are Essential for Meiosis and the Expression of Meiotic-Specific Genes
Source: PLoS Genet. 2017 Jan 9;13(1):e1006541. doi: 10.1371/journal.pgen.1006541 (PMC5261807; doi:10.1371/journal.pgen.1006541)
Supplement: S3 Table — (PDF) [file pgen.1006541.s010.pdf]

Table S3. List of plasmids.

Name	Description	Backbone	Source
pJG75	GST Sc Bdf1 Bd1 (residues 132-263)	pGEX4t1	This study
pJG76	GST Sc Bfd1 Bd2 (residues 317-430)	pGEX4t1	This study
pJG77	GST Bdf1 Bd1-Y187F	pJG75	This study
pJG78	GST Bdf1 Bd2-Y354F	pJG76	This study
pJG96	GST Brd4 Bd1 (residues 22-204)	pGEX4t1	This study
pJG109	GST Bdf1 Bd2-Y338W	pJG76	This study
