# Supplementary material for: Bdf1 Bromodomains Are Essential for Meiosis and the Expression of Meiotic-Specific Genes
Source: PLoS Genet. 2017 Jan 9;13(1):e1006541. doi: 10.1371/journal.pgen.1006541 (PMC5261807; doi:10.1371/journal.pgen.1006541)
Supplement: S4 Table — (PDF) [file pgen.1006541.s011.pdf]

Table S4. List of primers.

Name	Usage	Description	Sequence	Source
prJG247	RT-qPCR	*IME1* ORF R	TTATAGATGAACCTCAGGTGCCCTCC	Govin et al, 2010 NAR
prJG248	RT-qPCR	*IME1* ORF F	GGTGGTGATTTTTCAACGTCGAAGGC	Govin et al, 2010 NAR
prJG249	RT-qPCR	*IME2* ORF F	CAAGCATTGATTTTTGCAGGTATAA	Govin et al, 2010 NAR
prJG250	RT-qPCR	*IME2* ORF R	CTGCGGGCAATTTTGTTGA	Govin et al, 2010 NAR
prJG605	RT-qPCR	*NDT80* ORF F	GGAAGAGGATACGCCTGTTATC	This study
prJG606	RT-qPCR	*NDT80* ORF R	GAGGTCCCACTTTGAACTGTAG	This study
prJG253	RT-qPCR	*DIT2* ORF F	TCGTCAAAATGTTGGAGCCC	Govin et al, 2010 NAR
prJG254	RT-qPCR	*DIT2* ORF R	TTGGAGAAACTACAGAAATGCTGTG	Govin et al, 2010 NAR
prJG578	RT-qPCR	*SMK1* ORF F	CCAACCAACCGTATAGTAAGTCCGTAG	This study
prJG579	RT-qPCR	*SMK1* ORF R	CGAGCATAGAATTCTGCAAGAATGCACC	This study
prJG580	RT-qPCR	*SSP1* ORF F	AAAAGTACGCAAAAGCAAGGGA	This study
prJG581	RT-qPCR	*SSP1* ORF R	CAGTATCTTCATGCTTTTCGCCT	This study
prJG251	RT-qPCR	*NUP85* ORF F	CAGCAAAGAGTTTTCTGCATACGTATCAGG	Govin et al, 2010 NAR
prJG252	RT-qPCR	*NUP85* ORF R	ATTAAACACTCTGTCATCACCTAAATCACGG	Govin et al, 2010 NAR
prJG883	qPCR-ChIP	*IME1* PR F	TGCTTCCCTTGTAGTTCGGTA	This study
prJG884	qPCR-ChIP	*IME1* PR R	ATCTTTAACAATACTACTGGTCGATTT	This study
prJG881	qPCR-ChIP	*IME2* PR F	TTTTCCAATATCACTCAACGAGAA	This study
prJG882	qPCR-ChIP	*IME2* PR R	ACAGCCACCTTAACCGACTG	This study
prJG877	qPCR-ChIP	*AMA1* PR F	GCGTCAATGCCTCTATGTCA	This study
prJG879	qPCR-ChIP	*AMA1* PR R	TCCAAGGATTAAAGTGTTCTTTGAG	This study
prJG879	qPCR-ChIP	*NDT80* PR F	TACTTCCGCGGCTATTTGAC	This study
prJG880	qPCR-ChIP	*NDT80* PR R	CAACCGTTCTGGAGCTTTGT	This study
prJG58	qPCR-ChIP	*IME2* ORF F	GCCTAAAAATGGTCACCAAAACTATAT	Govin et al, 2010 NAR
prJG59	qPCR-ChIP	*IME2* ORF R	GCCACTACACCTTGTGTCTTTGTT	Govin et al, 2010 NAR
prJG182	qPCR-ChIP	*DED1* F1	AAGAGATGGAGGAACGGGAAA	Govin et al, 2010 G&D
prJG183	qPCR-ChIP	*DED1* R1	CCACTTGCCACCTATCACCAC	Govin et al, 2010 G&D
prJG194	qPCR-ChIP	*GUA1* F	TATGCTGAGATGTTGCCATGC	Govin et al, 2010 G&D
prJG195	qPCR-ChIP	*GUA1* R	AGTCCAGCCCAACTCAGAGATC	Govin et al, 2010 G&D
prJG929	qPCR-ChIP	F IntV	TAAGAGGTGATGGTGATAGGCGT	Wyce et al, 2007 Mol Cell
prJG930	qPCR-ChIP	R IntV	CCCTCGGGTCAAACACTACAC	Wyce et al, 2007 Mol Cell
